# Exploring mobility in Italian Neolithic and Copper Age communities

**DOI:** 10.1038/s41598-021-81656-z

**Published:** 2021-01-29

**Authors:** Flavio De Angelis, Maura Pellegrini, Cristina Martínez-Labarga, Laura Anzivino, Gabriele Scorrano, Mauro Brilli, Francesca Giustini, Micaela Angle, Mauro Calattini, Giovanni Carboni, Paola Catalano, Emanuela Ceccaroni, Serena Cosentino, Stefania Di Giannantonio, Ilaria Isola, Fabio Martini, Elsa Pacciani, Francesca Radina, Mario Federico Rolfo, Mara Silvestrini, Nicoletta Volante, Giovanni Zanchetta, Lucia Sarti, Olga Rickards

**Affiliations:** 1 Centre of Molecular Anthropology for Ancient DNA Studies; Department of Biology, University of Rome “Tor Vergata”, Via della Ricerca Scientifica 1, 00133 Rome, Italy; 2 Thermo Fisher Scientific, Strada Rivoltana 4, 20053 Rodano, MI Italy; 3 Research Laboratory for Archaeology and the History of Art, Dyson Perrins Building, South Parks Road, Oxford, OX13QY UK; 4 Istituto di Geologia Ambientale e Geoingegneria (IGAG), CNR, Area della Ricerca di Roma RM1. Via Salaria km 29,300, Monterotondo Stazione, 00015 Rome, Italy; 5 Istituto Autonomo Villa Adriana e Villa d’Este, Piazza Trento, 5, 00019 Tivoli, RM Italy; 6 Soprintendenza Speciale Archeologia, Belle Arti e Paesaggio di Roma, Piazza dei Cinquecento, 67, 00185 Rome, Italy; 7 Dipartimento di Scienze Storiche e dei Beni Culturali, Siena University, Via Roma 56, 53100 Siena, Italy; 8 Dipartimento di Scienze dell’Antichità, Sapienza University of Rome, P.le Aldo Moro, 5, 00185 Rome, Italy; 9 Soprintendenza Archeologia, Belle Arti e Paesaggio dell’Abruzzo, Via degli Agostiniani 14, 66100 Chieti, Italy; 10 Collaborator of Soprintendenza Archeologia, Belle Arti e Paesaggio dell’Abruzzo, Via degli Agostiniani 14, 66100 Chieti, Italy; 11 Istituto Nazionale di Geofisica e Vulcanologia, Sezione di Pisa, Via Cesare Battisti, 53, 56125 Pisa, Italy; 12 Dipartimento di Storia, Archeologia, Geografia, Arte e Spettacolo, Florence University, Via S. Egidio 21, 50122 Florence, Italy; 13 Former Soprintendenza Archeologia, Belle Arti e Paesaggio di Firenze, Pistoia e Prato, Florence, Italy; 14 Soprintendenza Archeologia Belle Arti e Paesaggio per la Città Metropolitana di Bari, Via Pier l’Eremita, 25, 70122 Bari, Italy; 15 Dipartimento di Storia, Patrimonio culturale, Formazione e Società, University of Rome “Tor Vergata”, Via Columbia 1, 00133 Rome, Italy; 16 Former Soprintendenza per i Beni Archeologici delle Marche, Via Birarelli 18, 60100 Ancona, Italy; 17 Dipartimento di Scienze Della Terra and Centro Interdipartimentale per lo studio dell’Impatto dei Cambiamenti Climatici (CIRSEC), University of Pisa, Via S. Maria 53, 56126 Pisa, Italy; 18 Present Address: Lundbeck Foundation GeoGenetics Centre, GLOBE Institute, University of Copenhagen, Øster Voldgade 5-7, 1350 Copenhagen, Denmark

**Keywords:** Archaeology, Archaeology

## Abstract

As a means for investigating human mobility during late the Neolithic to the Copper Age in central and southern Italy, this study presents a novel dataset of enamel oxygen and carbon isotope values (δ^18^Oca and δ^13^Cca) from the carbonate fraction of biogenic apatite for one hundred and twenty-six individual teeth coming from two Neolithic and eight Copper Age communities. The measured δ^18^Oca values suggest a significant role of local sources in the water inputs to the body water, whereas δ^13^Cca values indicate food resources, principally based on C_3_ plants. Both δ^13^Cca and δ^18^Oca ranges vary substantially when samples are broken down into local populations. Statistically defined thresholds, accounting for intra-site variability, allow the identification of only a few outliers in the eight Copper Age communities, suggesting that sedentary lifestyle rather than extensive mobility characterized the investigated populations. This seems to be also typical of the two studied Neolithic communities. Overall, this research shows that the investigated periods in peninsular Italy differed in mobility pattern from the following Bronze Age communities from more northern areas.

## Introduction

The complex interweaving of cultures associated with human mobility has traditionally represented a jigsaw puzzle for our understanding of the biological, cultural, and social evolution of past populations^1^. Unfortunately, population dynamics and mobility cannot easily be inferred from the archaeological record alone^2^ since the simple act of people moving from one place to another does not necessarily leave behind archaeological traces^3^. Even though mobility could have been a critical element of foragers’ strategies for resource exploitation^4,5^, it could also underlie other fundamental factors, such as social complexity and population dynamics.

The transition between the Neolithic and the Copper Age (dating around 4th millennium BCE in Italy) marked a tipping point for many aspects of peoples' lifestyles. This transition was accompanied by changes in settlement structures, subsistence strategies, cultural assemblages, and mortuary practices^6–8^.

Farming technologies were refined, and metallic ores such as copper started to be mined and used to produce artefacts in the Copper Age. The introduction of metal processing in the Copper Age led to a significant increase in the commercial trade^9^ and was complemented by the onset of a complex trade network associated with the movement of people^9–13^.

Archaeological evidence of goods movements can be integrated with the information retained in the human skeletons. Carbon and nitrogen isotope analysis of bone collagen for dietary reconstruction can provide information on subsistence strategies in human populations, though only rarely shifts in dietary habits have been used to support hypothesis concerning human mobility^14–16^. This is mainly associated with issues of collagen turnover and renewal in living organisms. Conversely, tooth enamel forms during the first years of life, becomes biologically inert, and does not undergo chemical modifications as individual ages^17^, recording the isotopic composition of dietary inputs during the early stages of life^18^. Such isotope fingerprint is inherited from the local environment. For this, isotope measurements in tooth enamel are useful for the identification of local and foreign individuals in an area. These investigations, in combination with other archaeological evidences^19^, prove valuable in addressing questions on human mobility.

Despite the plentiful archaeological surveys, the analysis of settlements and burial grounds in central and southern Italy has not yet provided a coherent picture of mobility dynamics for the Neolithic and Copper Age people. Archaeologists have often interpreted the transitions in typological structures of artefacts and the differences in funerary practices as reflecting a population shift due to mobility. This has been demonstrated in northern Italy but remains questionable in the central and southern Italian peninsula^20^ and reference therein. Isotope studies on human skeletons therefore represent valuable adjuncts to the few studies of mobility in Italy from the Neolithic to the Bronze Age^21–25^.

This paper aims at providing a new set of human tooth oxygen and carbon isotope measurements from Neolithic (Mora Cavorso, MC, and Galliano Palagiano, PA) and Copper Age (Buca di Spaccasasso, SS, Fontenoce di Recanati, FR, Celano Pratovecchio, CE, Osteria del Curato-Via Cinquefrondi, OC, Casetta Mistici, CM, Torre della Chiesaccia, TC, Pantano Borghese, PB, Grotta Nisco, GN) settlements/funerary areas (Table 1, Supplementary Figure S1)^26^. The archaeological record for those communities has suggested some mobility dynamics. Pottery typological heterogeneity and a variety of funerary practices, which could be consistent with imported items or deposition cultures developed with increased people mobility, characterized most of the archaeological contexts (Supplementary Information). However, the degree of mobility in each community cannot be assessed through the sole archaeological data, which could reflect only cultural diffusion. Indeed, the isotopic signatures provide a reliable disclosure of individuals’ mobility patterns, representing a valuable contribution to the comprehensive characterization of the Italian Neolithic and Copper Age communities.

**Table 1:** Radiocarbon dates and sample size of the sites. The dates reported are referred to as the maximum time range so far obtained for the sites.

Site (province)	Code	Date	Analyzed samples	Estimated individuals
Mora Cavorso (Rome)	MC	6275 ± 45 BP ÷ 6405 ± 35BP	9	28
Galliano-Palagiano (Taranto)	PA	5642 ± 50 BP ÷ 5448 ± 50 BP	9	9
Grotta Nisco (Bari)	GN	4723 ± 50 BP ÷ 4674 ± 50 BP	7	19
Celano Pratovecchio (L’Aquila)	CE	Not available	3	3
Fontenoce di Recanati (Macerata)	FR	4882 ± 62 BP ÷ 4478 ± 68 BP	18	32
Buca di Spaccasasso (Grosseto)	SS	4831 ± 50 BP ÷ 3506 ± 55BP	50	61
Casetta Mistici (Rome)	CM	4797 ± 45 BP ÷ 4240 ± 50 BP	7	15
Torre della Chiesaccia (Rome)	TC	4129 ± 45 BP	4	10
Osteria del Curato-Via Cinquefrondi (Rome)	OC	4865 ± 60 BP ÷ 3740 ± 70 BP	16	35
Pantano Borghese (Rome)	PB	4129 ± 45BP ÷ 3909 ± 55 BP	3	14

Overall, the sites cover about 2000 years and the studied samples provide a glimpse of human mobility in peninsular Italy for the addressed periods.

Our collection includes all the available skeletal material for these periods in the considered geographical areas, as granted by the Institutions involved in the project. Indeed, this investigation might be slightly biased due to specific population features but, despite some potential cultural peculiarities, our research aims to provide enamel oxygen and carbon isotopic signatures for these prehistoric Italian communities and therefore generate a novel cross-sectional overview of population dynamics and the extent of individual mobilities at the time. Future studies will likely implement our preliminary research once new sample collections are available.

### Scientific background

Several isotopic markers are used in palaeo-mobility research^27,28^. The most applied are oxygen and strontium isotopes from tooth enamel^28–35^, while the isotopes of other elements are seldom applied for this topic^32,35–38^. Mobility research via oxygen isotopes relies on site-specific isotopic signatures to gauge where people might have lived during the period of skeletal mineralization. Oxygen isotopes can be extracted from enamel, dentine, or bone samples. Human tooth enamel forms during childhood and adolescence^38,39^ and remains unaltered through adulthood. Tooth dentine grows incrementally during tooth formation with negligible remodeling later in life^40^, even though secondary and tertiary dentine keeps forming after tooth completion, making this tissue not completely isotopically inert. Bone tissue is instead continuously remodeled in life.

Individuals develop typical isotopic signatures based on the composition of foodstuff and water they consume. If dietary supplies are local, body signatures should reflect the isotopic fingerprinting of the regions where people live^41,42^. The isotopic features of the environment are in turn related to typical parameters such as temperature and precipitation, which influence in different ways both the carbon and the oxygen isotopes expressed in vegetation and drinking waters^43^. Oxygen enters the body water through drinking, eating, and breathing, and it is partially removed with respiration and discarded liquids and vapor^44–46^. Despite a metabolic fractionation between body water and tissues, oxygen isotopes in biological tissues are related to the water inputs to the diet and can thus be used to investigate human compatibility to a certain area^47^. Carbon isotopes reflect the local vegetation and can discern plants following C_3_ versus C_4_ metabolic cycles, as well as the consumption of marine resources^48^, as this information is transferred to the tissues of organisms feeding on those resources.

Thus, isotopes in skeletal remains are suitable proxies to investigate mobility and can assist in disentangling larger bioarchaeological questions at the community level^42^. This approach is however not devoid of complications: for example, a local signature can overlap with regions characterized by comparable environment^43,49^ or the same can be altered by food and drink manipulation.

Oxygen isotope values can be extracted from two components of biological apatite: carbonate (CO_3_) and phosphate (PO_4_); their isotopic signatures will hereafter be indicated as δ^18^Oca and δ^18^Op, respectively^49–54^. The relationship between δ^18^Op and δ^18^Oca with drinking water in humans is well established^44,53,55–57^. While measurements of δ^18^Oca is a robust and cost-effective method, which is often preferred over δ^18^Op measurements since it comes with the carbon isotope information (δ^13^Cca), the carbonate fraction of bioapatite is also more prone to postmortem alteration than the phosphate fraction^53,58^, for which extreme care has to be taken in its interpretation. Once converted to drinking water (δ^18^Odw), measured skeletal δ^18^Oca values can be compared with local mapped ground- or meteoric-water isotope values^59,60^, making it possible to evaluate if an individual signature is consistent with that expected for local people. However, the isotopic features of rainwaters in an area depend on local climate conditions, that could change through time, and rainwater is often not the sole source of drinking water for humans. Thus, the conversion of oxygen isotopes from skeletal items to environmental waters, is not straightforward^57,61^.

Alternative interpretative methods are based on geostatistical elaboration of skeletal δ^18^O values to assess the expected biological compositions for a specific area^34,35,62^. In this paper, we employed both approaches.

Some further complexities in isotopic mobility studies are associated to: different individual dietary habits (cooking, infusing, poaching); potential consumption of modified or imported water sources (e.g., wine, beers, etc.); drinking waters coming from further away (rivers) or that may have undergone extensive evaporations (lakes), and metabolic effects^47,50,63–65^.

Additionally, mineralized tissues such as tooth enamel might record an ^18^O enrichment due to breastfeeding until weaning starts, with later forming tissues scoring values closer to adult ranges^64–68^. However, this enrichment is not systematically observed, and may be blurred in many archaeological records because the collections could be composed of individuals whose breastfeeding was completed at different ages, or that may have grown up in slightly different climatic conditions. Therefore, the possibility of an increase in δ^18^Oca in juvenile teeth must be considered.

To summarize, although the isotopic approach to mobility is challenging, it can provide valuable insights into past human movements and interactions.

## Results

Oxygen isotope values (δ^18^Oca reported vs. V-SMOW^69^) and carbon isotope values (δ^13^Cca vs. V-PDB) were measured in the carbonate fraction of tooth enamel apatite of 126 individuals pertaining to 10 funerary contexts in central and southern Italy (Table S3).

**Figure 1:**
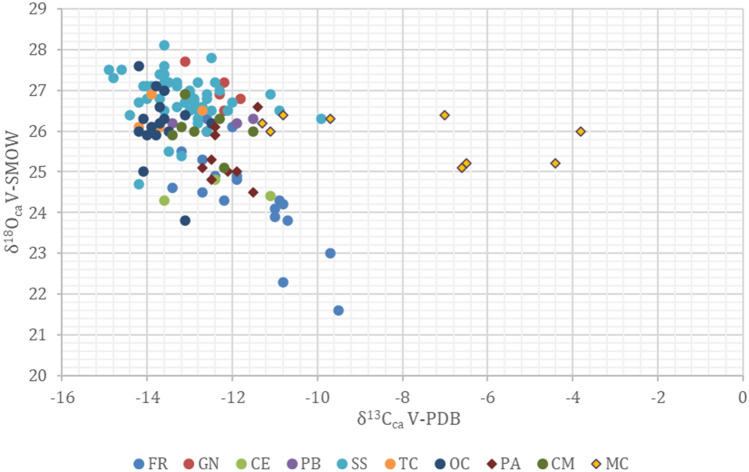
Bivariate plot of oxygen and carbon isotope values for the studied individuals. Samples coming from different locations are indicated with symbols of different colors. Different periods are reported with different symbol shapes: Dots = Copper Age individuals, Rhombus = Neolithic individuals. SS = Buca di Spaccasasso; FR = Fontenoce di Recanati; CM = Casetta Mistici, OC = Osteria del Curato-Via Cinquefrondi; TC = Torre della Chiesaccia, PB = Pantano Borghese; GN = Grotta Nisco; CE = Celano Pratovecchio; MC = Mora Cavorso; PA = Galliano-Palagiano.

Most of the samples cluster in a range from 24‰ to 28‰ in δ^18^O and from about − 15 to − 11‰ in δ^13^Cca (Fig. 1). Some samples from Mora Cavorso (MC) and Fontenoce di Recanati (FR) show a shift towards more ^13^C-enriched values.

Oxygen isotopes show a difference between the individuals from Fontenoce di Recanati (FR) and Celano Pratovecchio (CE), with typically lower δ^18^O values, and those from the other burial sites here investigated.

**Figure 2:**
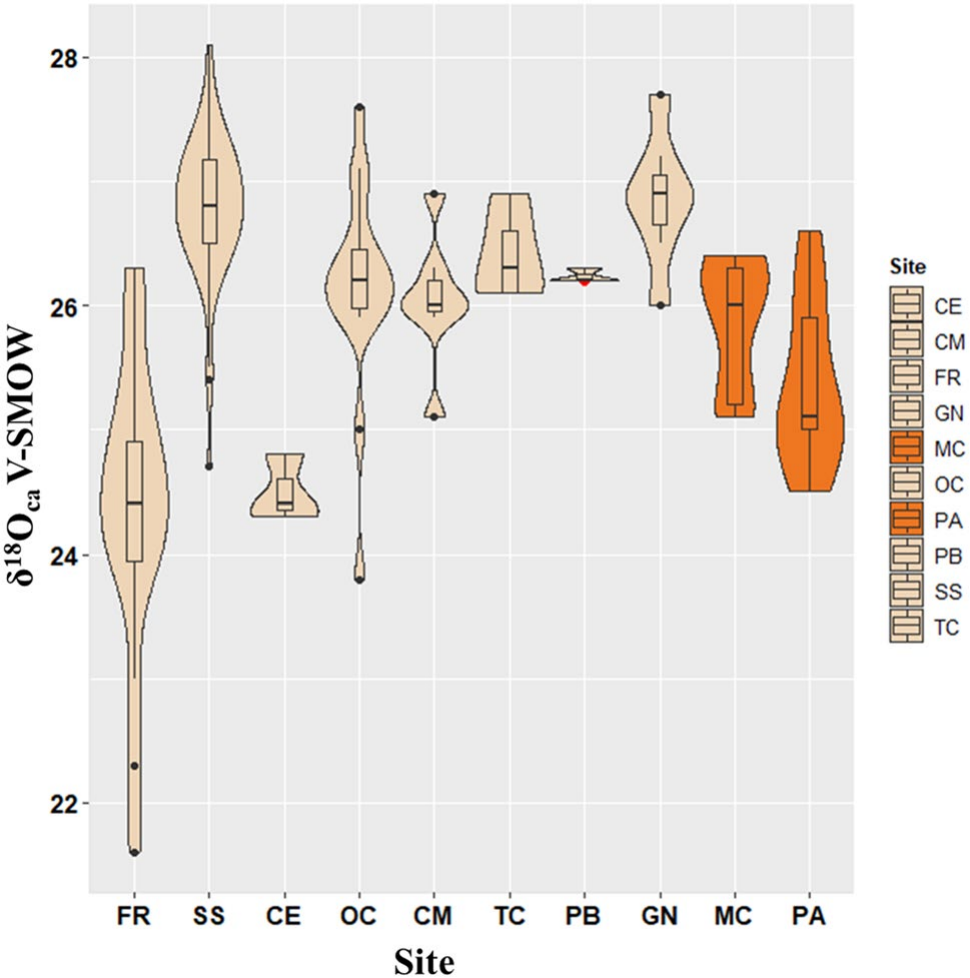
Violin plots for the oxygen isotope values (δ^18^O_ca_) of the Copper Age (dark-ivory) and Neolithic (orange) populations. The Copper Age and Neolithic sites are arranged from left to right according to decreasing latitude. The top and bottom limits of the embedded boxes indicate the third and the first quartiles, while the middle lines are the Medians. The top and bottom whiskers cover the statistical range for the population, and single dots represent individual outliers. SS = Buca di Spaccasasso; FR = Fontenoce di Recanati; CM = Casetta Mistici, OC = Osteria del Curato-Via Cinquefrondi; TC = Torre della Chiesaccia, PB = Pantano Borghese; GN = Grotta Nisco; CE = Celano Pratovecchio; MC = Mora Cavorso; PA = Galliano-Palagiano.

The Neolithic sites of Mora Cavorso (MC) and Galliano-Palagiano (PA) show intermediate δ^18^O values, with the former showing similar results to the nearest Copper Age sites (Osteria del Curato-Via Cinquefrondi, OC, Casetta Mistici, CM, Torre della Chiesaccia, TC, Pantano Borghese, PB); Galliano-Palagiano (PA) has values lower than the nearest Copper Age site in Apulia (Grotta Nisco, GN) (Fig. 2).

**Figure 3:**
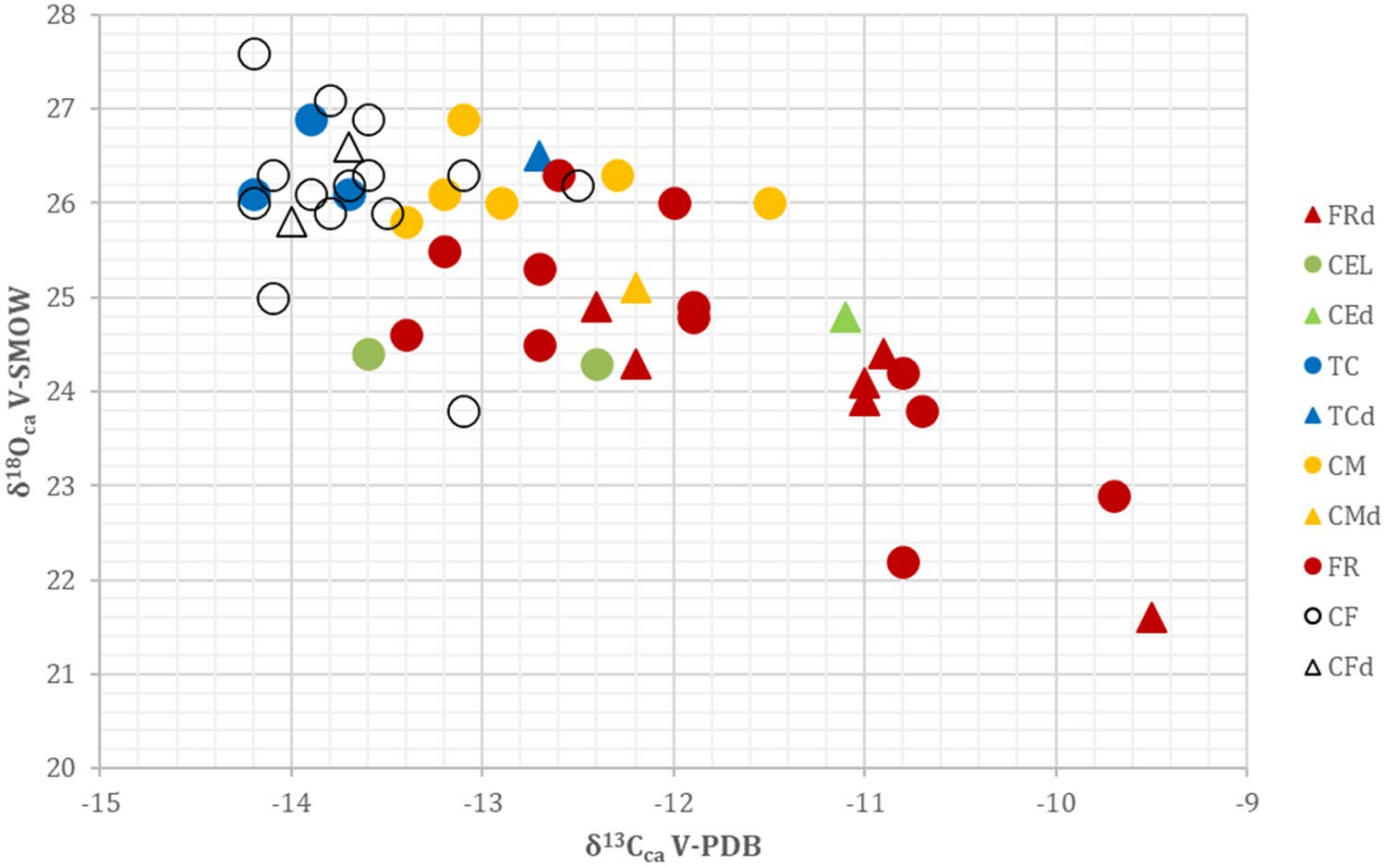
Bivariate plot of oxygen and carbon isotope values for deciduous and permanent teeth. Samples coming from different locations are indicated with different colors. Dots = Permanent teeth, Triangle = Deciduous teeth. FR = Fontenoce di Recanati; CM = Casetta Mistici, CF = Osteria del Curato-Via Cinquefrondi; TC = Torre della Chiesaccia,CE = Celano Pratovecchio.

Since breastfeeding could affect the δ^18^O values in our investigated teeth, systematic ^18^O enrichment^66^ in deciduous teeth and M1s was checked. Results were compared to enamel values from permanent teeth, which should be unhampered or minimally affected by the breastfeeding effect. All the values obtained from deciduous teeth fall within the range of adults or possibly weaned people in every site, and no systematic offset is detected (expected on the range of 0.5–2‰^64^), even though the limited sample size could partially mask this phenomenon (Fig. 3).

Measured oxygen isotope values were converted to environmental waters^57^ to assess general consistency between the bioapatite record of each group with local water sources, and to compare the ranges with those of (current) precipitations in the geographic areas of recovery. The results of these conversions are reported for each site in Table S1.

The individuals from Buca di Spaccasasso (SS, n = 50), located near the Tyrrhenian coast (southern Tuscany), show the highest δ^18^O values recorded in this study. These values have a mean of 26.8 ± 0.6 ‰, and converted to environmental water values they give an interval of − 6.0 ± 0.9 ‰ (Table S1), consistent with modern waters for the area, ranging between − 5 and − 6‰^59,60^.

Human teeth from Grotta Nisco (GN, n = 7) show similarly high δ^18^O values (26.9 ± 0.5 ‰), which converted to environmental waters give values of − 6.0 ± 0.9‰, (Table S1) consistent with modern local precipitations (− 6 to − 7‰^72,73^).

The Copper Age sites of Fontenoce di Recanati (FR) and Celano (CE) overlap, with mean δ^18^O of 24.4 ± 1.2‰ (n = 18) and 24.5 ± 0.3‰ (n = 3), respectively. The converted values from Celano are consistent with those of modern precipitations in the area (− 9.7 ± 0.4‰) but those of Fontenoce seem to be lower compared to the range of modern precipitations (− 9.9 ± 1.9‰)^59,60^.

**Figure 4:**
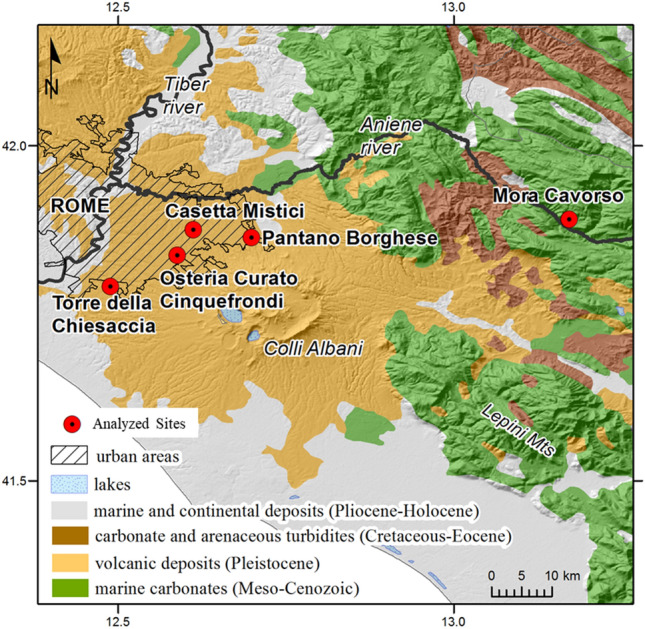
Location of the Roman sites. The geological features are shown in colors. Line-shading covers the current urban area of Rome. Geological units are from SGI-ISPRA geoportal (available at http://sgi.isprambiente.it/geoportal/catalog/main/home.page); hillshade was created using a GTOPO30 (Global 30 arc-second elevation) Digital Elevation Model, from the USGS database (accessible at ) with a resolution of 30″ × 30″ (about 1 km); the urban area of Rome is from ISTAT geoportal (available at https://www.istat.it/it/archivio/222527).

Samples from Osteria del Curato-Via Cinquefrondi (OC), Casetta Mistici(CM), Torre della Chiesaccia (TC), and Pantano Borghese (PB), all coming from Rome area, overlap and have similar δ^18^O medians (Pantano Borghese, PB-Torre della Chiesaccia, TC, n = 2; T-test − 0.73; *p* = 0.25; Osteria del Curato-Via Cinquefrondi (OC), Casetta Mistici (CM) and the Pantano Borghese-Torre della Chiesaccia group, n = 16;7;7 Kruskal–Wallis H = 1.85; *p* = 0.40). The proximity of these sites (Fig. 4), their contemporaneity, and similarity in isotopic results make them suitable for grouping together for statistical analysis. The median of “Rome Copper Age group” for δ^18^O is 26.2 ‰ ± 0.7 (n = 30), and the, converted to environmental waters^57^, gives values averaging − 7.1‰ ± 1.1, consistent with the − 6 to − 7‰ range for modern waters in the area^59,60,74–76^. Mora Cavorso samples have a converted mean value for environmental water slightly lower than the Roman sites (− 7.5 ± 0.9 ‰).

The samples of the Neolithic site of Galliano-Palagiano (PA) have an average value of 25.4 ± 0.7‰ (n = 9). The calculated environmental water (− 8.3 ± 1.1 ‰) is significantly lower than the expected value for the site^59,60^.

**Figure 5:**
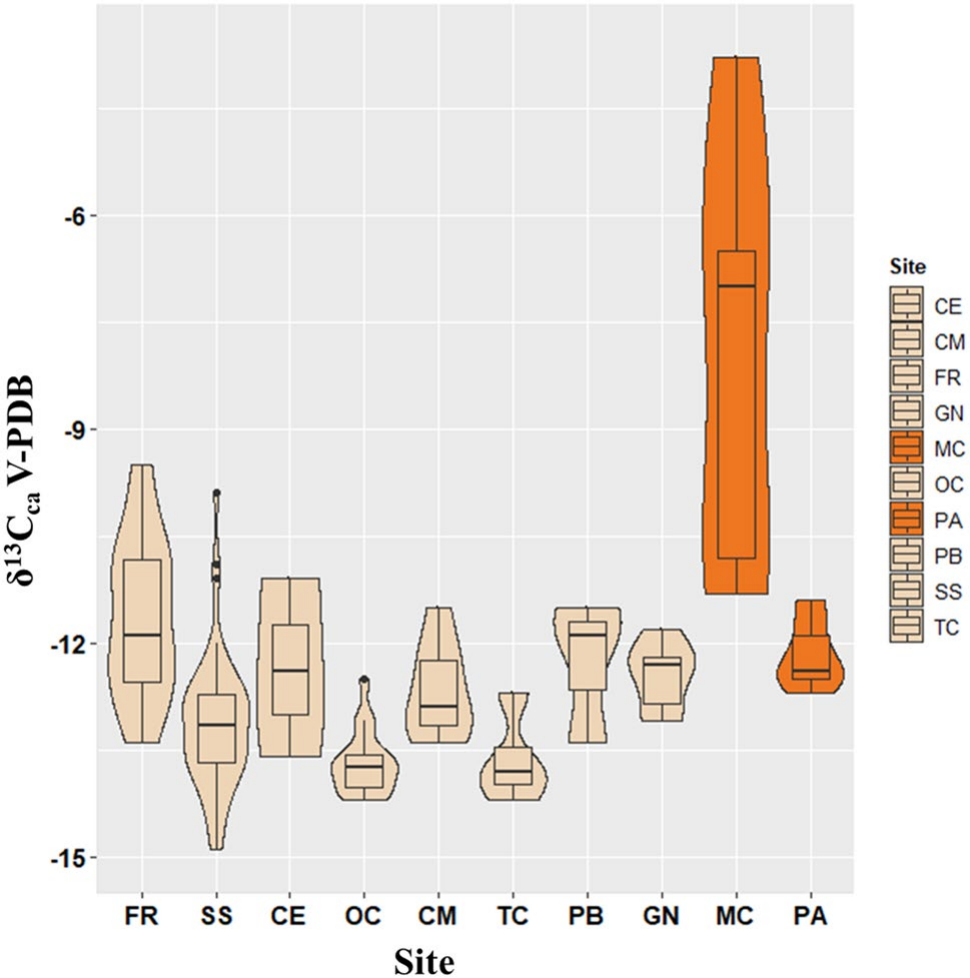
Violin plots for the carbon isotope values (δ^13^C_ca_) of the Copper Age (dark-ivory) and Neolithic (orange) populations. The Copper Age and Neolithic sites are arranged from left to right according to decreasing latitude. The top and bottom limits of the embedded boxes indicate the third and the first quartiles, while the middle lines are the Medians. The top and bottom whiskers cover the statistical range for the population, and single dots represent individual outliers. SS = Buca di Spaccasasso; FR = Fontenoce di Recanati; CM = Casetta Mistici, OC = Osteria del Curato-Via Cinquefrondi; TC = Torre della Chiesaccia, PB = Pantano Borghese; GN = Grotta Nisco; CE = Celano Pratovecchio; MC = Mora Cavorso; PA = Galliano-Palagiano.

Carbon delta values are mostly restricted to a range of − 14 to − 11‰ in all sites (Fig. 5); some samples from Mora Cavorso (MC), however, diverge substantially, showing values as high as − 4‰.

## Discussion

The samples investigated in this study cover a timeframe of several centuries and possibly various environmental conditions. Provided that the calculated environmental water (Table S1) values are comparable with modern rainwaters, there is a correspondence between water and skeletal values in most cases, even though it must be considered that indeed some climate fluctuations have characterised the Middle Holocene^70,71^, and these likely had effects on the rainwater oxygen isotope ratios. With this in mind, we could observe that converted values from the samples of Buca di Spaccasasso (SS), Grotta Nisco (GN) and all the sites scattered in the Roman area are consistent with modern precipitations; the lower values recorded at Celano Pratovecchio (CE) might instead be associated to the location of this site on the Apennines mountain chain, with peaks higher than 2000 mt a.s.l.. These areas are typically characterised by low mean annual temperatures and consequent low δ^18^O values in precipitations^59,60,72,73^. The samples from Fontenoce di Recanati (FR) are also characterised by relative low oxygen isotope values, though this site is located at a much lower altitude than Celano (CE). It is however sited at the foothills of the range and it is reasonable to assume that the water sources for this site could have been springs or riverine waters fed by precipitations falling at higher elevations, similar to those available at Celano (CE).

It can also be assumed that, given the relative low values typical of the samples from the site of Galliano-Palagiano (PA), despite its location, the primary source of drinking water originated at higher elevations in the Apennines^77^. Clearly, the drinking water at this site compositionally differed from the one supplying the Copper Age community buried in the other Apulian site of Grotta Nisco (GN), for which the calculated δ^18^Odw is − 5.9 ± 0.8‰. A possible explanation for the observed difference, besides the use of different water resources, could be that they experienced different climatic conditions^70,78^.

Overall, the implication suggested by the results is that most of the populations investigated relied on local, unmodified water sources for their needs.

The attempt to identify non local people was pursued via an individual δ^18^O signature evaluation in comparison with that of the local population, assuming that the majority of the investigated skeletons represents resident people. The deciduous teeth values fall among adults δ^18^O values (Fig. 3), showing that despite stable oxygen isotope analysis from bulk sampling could be used empirically to reconstruct weaning ages, successful identification of this practice depend on multiple factors^63,79–83^ and does not systematically result in a detectable rise of the isotopic signature.

To estimate the presence of possible outliers in each site, we followed the discussion in Lightfoot and O’Connell^62^.

The whole range of δ^18^O values from tooth bioapatite at Buca di Spaccasasso (SS) is 3.4‰. If the MAD threshold is applied (MADQ3) (see^62^ and reference therein) (n = 50, Shapiro–Wilk test W = 0.95; *p* = 0.02), one sample can be spotted as an outlier (SPS_23p at 24.7‰) by all the four statistical evaluations and should be considered a newcomer.

SPS_25p (25.4‰), SPS_44p (28.1‰) and SPS11p (25.5‰) are outside the range obtained using 2‰ offset from the mean, but only SPS_25p is highlighted as a “not local” by 1,5IQR range.

The lowermost skeletal values are too depleted in ^18^O compared to the rest of the people from the same site: they result in converted values for the d18Odw of about -9.3‰ and, even considering the mathematical error associated with the conversion of 1–2‰^57,61^, they seem to be more pertinent to individuals from the highlands of the Apennine, the Alps, but also other regions further away in north-western Europe. These individuals seem more typical, for example, to the highlands of the Apennines or the Alps, but also to other regions further away in north-west Europe. The identification of their exact origin is impossible with the current available data.

The δ^13^Cca for the individuals identified as outliers does not differ substantially compared to the rest of the people from Buca di Spaccasasso (SS).

Grave goods with noteworthy typological features from the ossuary enclosure seem to indicate a deep similarity with some southern Tuscany and Conelle contexts in the Marche region. This evidence could suggest a mobility pattern involving both southern areas of Tuscany and upper Latium and the trans-Apennines areas such as the Marche region. A remarkable presence of items typologically inspired to the southern-Italian Laterza cultural complex, mainly set in Apulia, is also remarkable^84^. This link seems to be also suggested by dietary habits analyses^85,86^ that highlighted a common exploitation typology between people at Buca di Spaccasasso (SS) and the community buried in the Apulian site of Grotta Nisco (GN).

The δ^18^O range for Fontenoce di Recanati (FR) is 4.7‰, from 21.6 to 26.3‰ (Table 2). The application of the 3MADnorm threshold reduces the locality-range to 4.2‰ (from 22.3 to 26.5‰) (n = 18, Shapiro–Wilk test W = 0.95 *p* = 0.47). The range excluded FRN 6.1, set beyond the lower end of the range, while a second individual (FRN 19.1) sits at the boundary. These two individuals lie outside the locality range also for 1.5IQR and the 2‰ offset from the mean. The identified outliers were buried in multiple graves containing at least two individuals, even though the non local features are derived from only one member of each skeletal couple. FN6.1 was a baby buried in association with a specific funerary ceremony involving the deliberate slaughter of a dog. This kind of ritual process seems to be quite widespread in the Italian peninsula since the Neolithic^87,88^ and was also found in the Abruzzi Neolithic site of Grotta Continenza.

**Table 2:** Descriptive statistics for the investigated populations.

Site	n	δ^18^O_ca_‰ V-PDB				δ18O_ca_‰ V-SMOW				δ13C_ca_‰ V-PDB			
		Min	Max	Mean	Stdv (1σ)	Min	Max	Mean	Stdv (1σ)	Min	Max	Mean	Stdv (1σ)
Fontenoce di Recanati	18	− 9.0	− 4.5	− 6.4	1.2	21.6	26.3	24.4	1.2	− 13.4	− 9.5	− 11.6	1.1
Buca di Spaccasasso	50	− 6.0	− 2.7	− 4.0	0.6	24.7	28.1	26.8	0.6	− 14.9	− 9.9	− 13.1	0.9
Celano Pratovecchio	3	− 6.4	− 5.9	− 6.2	0.3	24.3	24.8	24.5	0.3	− 13.6	− 11.1	− 12.4	1.3
Osteria del Curato-Via Cinquefrondi	16	− 6.9	− 3.2	− 4.6	0.2	23.8	27.6	26.2	0.9	− 14.2	− 12.5	− 13.7	0.1
Casetta Mistici	7	− 5.6	− 3.9	− 4.7	0.5	25.1	26.9	26.0	0.5	− 13.4	− 11.5	− 12.7	0.7
Torre della Chiesaccia	4	− 4.7	− 3.9	− 4.4	0.4	26.1	26.9	26.4	0.4	− 14.2	− 12.7	− 13.6	0.6
Pantano Borghese	3	− 4.6	− 4.5	− 4.6	0.1	26.2	26.3	26.2	0.1	− 13.4	− 11.5	− 12.3	1.0
Grotta Nisco	7	− 4.8	− 3.1	− 3.9	0.5	26.0	27.7	26.9	0.5	− 13.1	− 11.8	− 12.5	0.5
Galliano-Palagiano	9	− 6.2	− 4.2	− 5.4	0.6	24.5	26.6	25.4	0.7	− 12.7	− 11.4	− 12.2	0.5
Mora Cavorso	9	− 5.6	− 4.4	− 4.9	0.5	25.1	26.4	25.9	0.5	− 11.3	− 3.8	− 7.9	2.9

FN19.1 was a man buried with an infant, with no evidence to be considered a newcomer.

Given the similarities in the environmental settings and the same δ^18^O patterns, all the samples from the Copper Age sites around Rome were grouped to enhance the statistical power.

The range of values is 3.8‰ (Table 2). The 1.5IQR reduces this range to 1.6‰ for considering local individuals (from 25.4 to 27‰). The application of Tukey’s 1.5IQR method allows us to detect 6 individuals (CF2.2, CF7.3, CF17, CF24, CF27, T1NEC) as atypical in the context of the site, and four of these (CF2.2, CF7.3, CF27, T1NEC) showed δ^18^O values outside of the MADQ3 range too^57^ (n = 30, Shapiro–Wilk test W = 0.87, *p* = 0.00). Three of those individuals pertain to Osteria del Curato-Via Cinquefrondi (OC CF2.2, CF7.3, CF27), and one from Casetta Mistici (CM T1NEC). Finally, by employing the 2σ deviation from the mean value, only one extreme individual from Osteria del CuratoVia Cinquefrondi (OC CF2.2) can be identified as an outlier, and the same is also obtained by setting the threshold at 2‰ from the mean. None of the identified outliers for δ^18^O have unusual δ^13^Cca values.

CF7.3 was a child secondarily buried in a “grotticella” tomb, which is typically related to the Rinaldone-style multiple burials in the Rome area, containing both synchronic and diachronic remains. CF7.2 was a teenager buried in the same tomb but whose oxygen isotope signature supports a local origin, thus the same multiple burial could have hosted people of various origins, synchronically buried or not.

CF2.2 (4032 ± 45 BP, cal. 2σ 2700–2460 B.C.) and CF27 (4030 ± 65 BP, cal. 2σ 2900–2300 B.C.)^89^ were young adults referred to the Ortucchio culture complex recovered in different areas of the Osteria del Curato-Via Cinquefrondi site (OC). This cultural frame could be related to a sort of modification of the pre-existing Laterza culture framework in Latium, due to the spread of Bell Beaker culture from northern regions towards central Italy^89^. This cultural modification seems to have been accomplished through contact among people carrying out substantial changes in ceramic production. CF24 and CF17 (3919 ± 45 BP, cal. 2σ 2500–2280 B.C.) could also be categorized as Ortucchio-related tombs, but their goods seem to distinguish their status. Indeed, CF24 owned a generic vase and a single used arrowhead close to the body, while CF17 was buried with a pottery bowl that was ritually broken and put close to the pelvis. The rest of the people buried with broken pots show these items placed by the side of the head, suggesting a unique feature for this woman, possibly of non-indigenous origin. However, the relatively high δ^18^O value could also be related to metabolic alterations as diffuse periostitis on her long bones and heavy alveolar resorption in the jaws are present^90^.

The non-local origin of sample T1 in Casetta Mistici (CM)^91^ was previously hypothesized due to the southern spread of the Rinaldone culture complex, a cultural prospect mainly based in Tuscany, Umbria, and Marche. The topographical scattering of that tomb is also remarkable because the T1 child was recovered in the burial ground located outside the settlement itself. Despite the early age at death, this individual does not show a clear indication for breastfeeding as he/she is characterized by an isotopic value depleted in ^18^O compared to the local range of people. This evidence seems to be remarkable for considering this child coming from elsewhere with his/her mother possibly not be represented in the analysed samples.

The sample size for each of the Neolithic sites is rather small and accordingly, the statistical elaborations are less powerful. However, their analysis is still of considerable interest due to the different chronologies with the previous sites and some peculiarities. The data at Galliano-Palagiano (PA) shows a range of 2.1‰ (Table 2) and, after setting the thresholds for the proposed indicators, we identify one putative newcomer (PAL10; detected by the MAD method). The mean oxygen isotopes signature for this site is quite different from what would be expected based on its location and this result could be consistent with the funerary usage of the site by people coming from the neighboring areas (Supplementary material). A similar scenario of low range mobility was also deduced by strontium isotope analysis in other Neolithic sites from northern Apulia^21^.

Mora Cavorso (MC) deserved a more in-depth approach as the carbon isotope values seem worth discussing. Generally, in this study, δ^13^Cca is consistent with a C_3_ plant-based diet. However, δ^13^Cca at Mora Cavorso (MC) ranges from − 11.1 to − 3.8‰. Apart from a few individuals (12, 11?, 13*, and possibly 11*, with − 11.1‰, − 11.3‰, − 10.8‰, and − 9.7‰, respectively), the rest of the people have unusually ^13^C enriched values (δ^13^Cca ranging from − 3.8‰ to − 7.0‰). These people with data at odd with the general trend might potentially represent individuals originated in other geographical areas, and therefore individuals that have relocated to this site after childhood.

At first glance, these unusual values could also be consistent with a contribution of C_4_ plants to the diet, even though the consumption of marine food cannot be ruled out^92^. This hypothesis is puzzling as C_4_ plants consumption in the Italian peninsula is assumed to have started in the Bronze Age^14^. Besides, the topographic location of Mora Cavorso (MC) seems incompatible with the exploitation of marine fish. The bioapatite results of Mora Cavorso (MC) are also incongruous with those from collagen of individuals from the same site, ranging from 19.7 to − 20.5‰, and thus suggesting a diet principally based on C_3_^93^. This holds true even taking into account that collagen and bioapatite δ^13^C may record different components of the diet, with the former^92^ reflecting mainly the protein fraction, and the latter the entire diet (carbohydrates, lipids, and proteins)^94^. Although the presence of C_4_ plants in the area is unrealistic, also tooth enamel of two herbivores (one sheep and one deer) investigated at this site gave similar results (δ^13^Cca: − 5.4‰ and − 6.8‰, δ^18^Oca: 25.6‰ and 25.2‰, respectively), while faunal collagen δ^13^C data^93^ ranges from − 18.6 to − 23.6‰, thus suggesting a variable diet. Therefore, the peculiar δ^13^Cca in some of the individuals at Mora Cavorso (MC) could also be primarily interpreted with a diet based on the consumption of ^13^C-enriched prays.

One of the possible explanations, however, is still that the protocol of pre-treatments have changed the pristine isotopic composition of these samples^95–97^, even though, in this case, the biological signature should have been modified by several per mils to reach the observed values, which seems rather unrealistic.

Diagenetic changes from secondary carbonate of the karst cave should also be assumed as a confounding factor.

**Figure 6:**
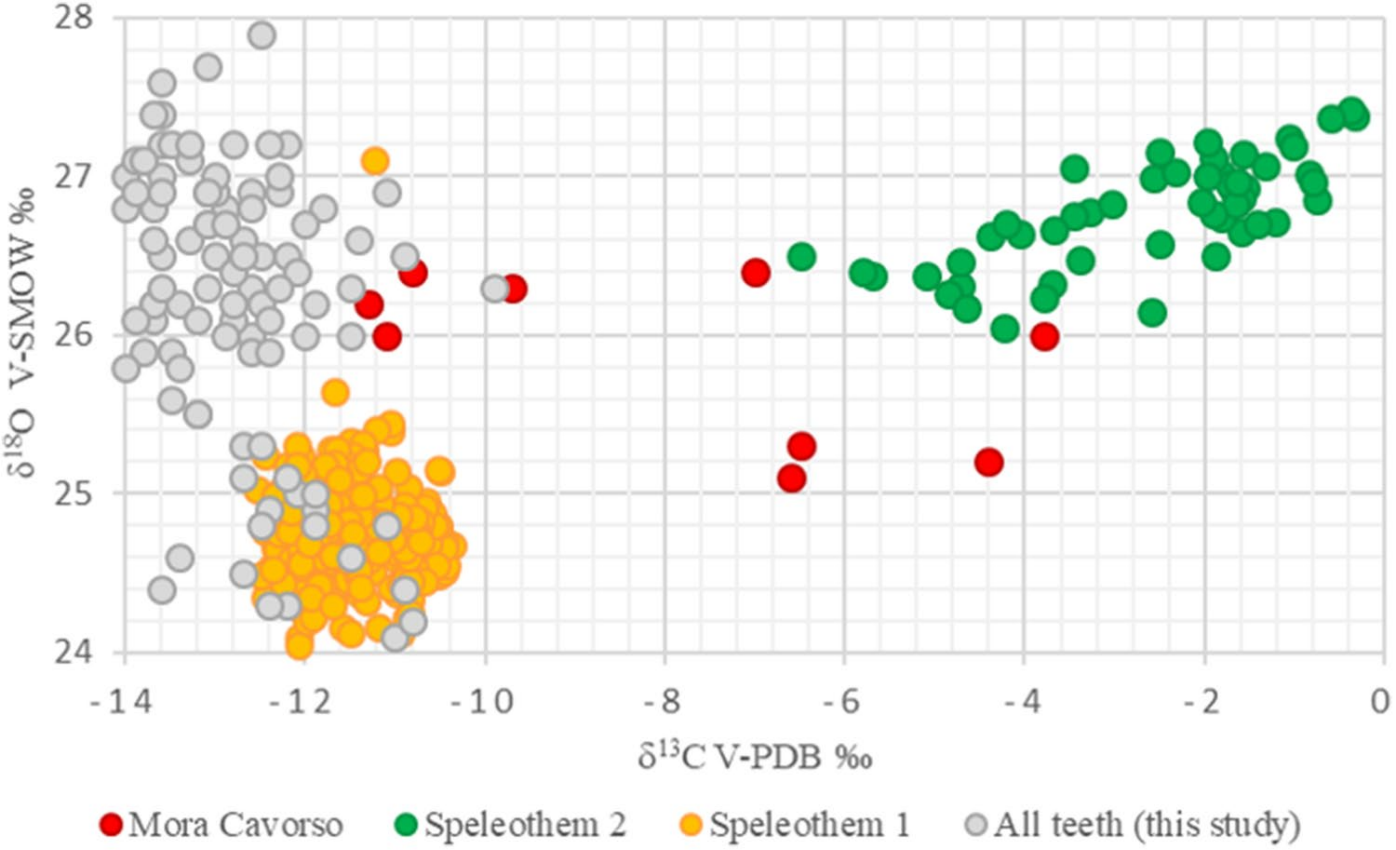
Bioapatite measurements plotted against the values of two speleothems sampled from Mora Cavorso (MC) cave. Speleothem 1: Speleothem formed during the MIS5, ca. 126 kilo years ago; Speleothem 2: Holocene speleothem. All teeth label refers to the analyzed teeth except for Mora Cavorso (MC) samples.

To check the occurrence of post-mortem carbonate contaminations of the Mora Cavorso (MC) samples, the bioapatite measurements were plotted against the carbonate values of two speleothems sampled from the same cave (Supplementary Table 4; Fig. 6), one formed during the MIS5 ca. 126 kya) and the other one formed during the Holocene, and representative of the tufa covering the archaeological sequence.

The teeth from Mora Cavorso (MC) lie between the carbonate composition of the two speleothems in the cave. They are also between the carbonate composition of most of the other teeth analysed, representative of human variability at the time, and the Holocene karst precipitations, on which the archaeological record is embedded. This evidence suggests that the chemistry of the teeth might have been modified by circulating fluids in the cave, with a critical impact on the carbon isotope composition. The δ^18^O in the teeth have instead a similar range to most of the investigated teeth, and any interaction with a fluid of similar composition would not be so evident.

None of the explanations above can fully justify the results from Mora Cavorso (MC) at this stage, therefore, no reliable hypothesis on the mobility pattern of this population can be completed.

Compared to the oxygen isotope results for the Bronze Age communities from northern Italy^22–25^, our study shows little evidence of extensive mobility in the central and southern Italy in earlier communities. This is consistent with the sulphur data of Bronze Age central Italian samples, where only a few individuals were considered as newcomers at Grotta dello Scoglietto^23^. The mobility patterns of Neolithic and Copper Ages people in these areas seem far from those related to social strategies of the northern Italian Bronze Age people who switched from a residential-based mobility during the Early Bronze Age to socially and politically motivated displacements with the progressive stabilization of the settlements^22^. Moreover, the data obtained in this study seems to support the idea that Neolithic and Copper Age people from central Italy were generally sedentary, and sporadic mobility was associated with a few individuals. Our results support the observations provided for Neolithic communities in Apulia, where a sedentary lifestyle was preferred despite the widespread of social and economic interactions and the presence of specific sites where people coming from elsewhere were buried^21^.

## Conclusions

This study presents the first set of tooth bioapatite isotope data from Italian Copper Age and Neolithic sites. The δ^18^O from the carbonate fraction of bioapatite shows significant differences between people buried at different funerary areas of the Italian peninsula but indicating a good relationship between the measured values and the local water sources. Overall, the number of outliers identified is quite small, suggesting that mobility was rather sporadic in the Copper Age and perhaps linked to small groups, and this seems to be even more sporadic in the Neolithic communities.

Convincing outliers were identified at the Copper Age sites from Rome, Fontenoce di Recanati (FR) and Buca di Spaccasasso (SS). These results are consistent with the archaeological record, supporting contacts among people fostering the typological circulations in central and southern Italian Copper Age.

The lack of a comprehensive osteological characterization of each individual, mainly due to a combination of poor skeletal preservation and the nature of bone assemblage, prevents the demographic assessment of the putative identified newcomers in some sites. Although the relatively small number of individuals might limit the robustness of the observations, this study represents a valuable contribution for investigating the mobility patterns of Italian prehistoric communities, which is a crucial topic in Italian archaeology. An increase in sample size is mandatory to frame the wide archaeological and anthropological scenario properly; a supplementary genomic analysis would also help to identify the origins of the recognized newcomers.

## Methods

One hundred and twenty-six teeth from people buried at ten central and southern Italian sites of Neolithic (Galliano Palagiano, PA, in Apulia, and Mora Cavorso, MC, in Latium) and Copper Age (Buca di Spaccasasso, SS, in Tuscany, Fontenoce di Recanati, FR, in Marche, Osteria del Curato-Via Cinquefrondi, OC, Casetta Mistici, CM, Torre della Chiesaccia, TC, and, Pantano Borghese, PB, in Latium, Celano Pratovecchio, CE, in Abruzzi and Grotta Nisco, GN, in Apulia) were collected and analyzed for this study (Table 1). Details of the funerary contexts are in Supplementary Information section. Permission for managing the materials and their use in this research were obtained by the formal commitment from each Superintendence, responsible for the anthropological remains.

The investigated teeth are primarily permanent M2, and comprise a range of other teeth, including some deciduous. The availability of skeletal items of choice is limited in the selected sites; therefore, to obtain some statistical representation of the investigated populations, it was decided to analyze all accessible teeth, regardless of the life-stage of the individuals they would represent. Accordingly, the analysis includes teeth formed at a very young age, potentially biased by the breastfeeding effect. However, it has been observed that, although there is a known trend in deciduous and early-forming teeth (such as incisors and M1) to be enriched in ^18^O with breastfeeding, such enrichment is not systematic^66^. Nevertheless, specific care was taken in interpreting these results and a comparison between deciduous and permanent teeth was carried out.

The description and archaeological background of each site are provided in the Supplementary material. Most of the sites suggest the presence of putative non-local migration due to the recovery of non-local items and various funerary practices. All the recovered skeletons at each site have been screened and the individual selection was driven by the presence of teeth and their good macroscopic preservation status. The basic demographic parameters of these specimens were marked according to classical anthropological methods^98,99^ and are provided in Supplementary Table S2. Only one tooth per individual was sampled. At Buca di Spaccasasso (SS), no anthropological evaluation could be performed due to the nature of the bone assemblage (see Supplementary material). Accordingly, only the right second molars from the mandibles were sampled for this site.

The identification of the dental elements for sampling was carried out by following odontological criteria^100^. Whenever possible, the second molar was selected, though in some cases different dental elements were chosen. Regardless the fact that different teeth might result in a slight bias since they represent various periods in a person’s life, we chose to sample as many individuals as possible for this isotopic study. Bulk sampling in each tooth at a fixed distance from the root-enamel junction in a consistent manner was performed^64,101^. Indeed, the sampling for permanent teeth was performed by drilling the buccal areas superior to the enamel-root junction (ERJ)^68^, and reference herein even though we are conscious of extensive discussion of methodological approaches that could mitigate inter-teeth sampling. This strategy allows us to collect data related to the post-weaning period for incisors, canines and first and second molars (3.5–6 years). Few premolars and third molars were included in the sampling even though the isotopic information provided could be related to the subsequent ontogenetic period.

Furthermore, few deciduous teeth were sampled to preliminarily investigate the role of confounding factors such as breastfeeding in mobility-related oxygen isotopes analysis.

Teeth were mechanically abraded and cleaned to remove soil and exogenous materials. They were then soaked in 0.1 M acetic acid for 2 h at room temperature to remove external diagenetic precipitates such as secondary carbonate^97,102^, followed by a rinsing step with deionized water and overnight drying at 37 °C. An aliquot of 25 mg/tooth of enamel was drilled and treated according to Sponheimer^103^: 1.8 ml NaOCl (2.5%) was added to the sample for 1 h and subsequently removed by three washing steps in deionized water. The powder was then treated with 1.8 ml acetic acid 0.1 M for 1 h and rinsed again three times. The lyophilization procedure returned a purified enamel powder to be analyzed by mass spectrometry. Approximately 2 mg of enamel powder was measured in duplicate along with three internal standards (MC-200, CaCO3—Merck CCM, and Solnhofen limestone—SLNF, calibrated against international standards NBS18 and NBS19) to normalize the raw δ^18^Oca and δ^13^Cca values to the V-PDB scale. Repeated analyses of internal carbonate standards (n ≫ 30) yield 1std dev < 0.1‰ for both δ^13^Cca and δ^18^Oca. An aliquot of the enamel powder for each sample was weighed and placed into empty vials and analyzed by a Thermo Scientific™ Gas Bench II connected to a Thermo Delta Plus isotope ratio mass spectrometer. The data obtained were normalized by a linear calibration equation derived from a plot of accepted versus measured values for the three aforementioned internal standards.

Oxygen isotope measurements were converted to δ^18^Oca V-SMOW according to IUPAC recommendations for oxygen isotope data^104^, by applying the following conversion equation^105^:

$${\delta^{18}}{\text{Oca}}\left( {\text{V - SMOW}} \right) = 1.03091*[{\delta^{18}}{\text{Oca(V - PDB)}}] + 30.91.$$

This also allows an easier comparison with previously published data from tooth carbonate in Italy and with phosphate oxygen isotope measurements produced from bioapatite, from which it is expected an offset of about 7–9‰^53,57,105,106^. Given, however, that this conversion could be associated with small mathematical errors, statistical analysis was performed on both the raw and the calibrated results, with no difference between the outcomes.

The equation provided in Chenery et al.^57^, was applied to convert teeth values to environmental water values (δ^18^Odw): δ^18^Odw = 1.59 * δ^18^Oca(V-SMOW) − 48.634.

Values are reported by violin plots: this type of plot, similar to other types of box plots, make it possible to visualize the distribution of data in a population, with the box embedded inside indicating the lower and upper quartile (i.e. the interquartile range, IQR) and the middle line indicating the median for the distribution of the values. The lower and upper whiskers indicate the spread of values outside the interquartile range (i.e. the values that are below the 25% and above the 75% mark). Single dots outside the whiskers represent the outliers in the population^107^.

Descriptive statistics and comparison tests have been performed by R v.3.6.1^108^, which makes it possible to draw the boxplot describing the variability of the data in each site and to perform the Shapiro–Wilk test to check the goodness of fit to the normal distribution of the dataset. The significance threshold was set equal to *p* = 0.05.

To estimate the presence of possible outliers in each site, we followed a statistical approach as discussed in Lightfoot and O’Connell^62^. Thresholds were set as: (a) 2σ deviation from the mean, (b) 3 times (MAD) (Median Absolute Deviation) from the median, for normal distribution of the data (MADnorm) or non-normal distribution of the data (MADQ3), (c) 1.5 Interquartile Range (IQR), and (d) 2‰ offset from the mean (Table 3). The statistical approach for the identification of outliers was applied only for those groups with a sufficiently high number of individuals (i.e., n ≥ 9). This limit was set to perform the statistical analysis in as many sites as possible, since sample size for each funerary context varies and generally comprise small communities. The validity of these results might however be questionable, since Lightfoot and O’Connell^62^ indicated 25 as the suitable sample size for this approach, a number hard to obtain in most archaeological Italian contexts for these periods.

**Table 3:** Modifying values for each community. Mora Cavorso was not considered for such analysis (see text for details).

	IQR	MAD_raw_	MAD_norm_	MAD_Q3_
Fontenoce di Recanati	0.9	0.5	0.7	–
Buca di Spaccasasso	0.7	0.3	–	0.5
Copper Age Rome	0.4	0.2	–	0.3
Galliano-Palagiano	0.9	0.3	–	0.4

Samples of the speleothems from Grotta Cavorso for stable isotope ratios were obtained with an air-drill 1 mm drilling bit. Stable isotope compositions (oxygen and carbon) were measured using an Analytical Precision AP2003 IRMS at the University of Melbourne on CO_2_ gas released by reaction with 105% H_3_PO_4_ at 70 °C. Results were normalized using an internal working standard (NEW1, Carrara Marble), calibrated against the international standards NBS18 and NBS19. Results are reported on the VPDB scale with the δ-notation in per mill (‰). Mean analytical uncertainties are 0.10‰ and 0.05‰ for δ^18^O and δ^13^C, respectively. Maps were generated using Esri ArcMap 10.4.1 software^109^.

## Supplementary information

Supplementary Information 1.
