# Supplementary material for: Exploring mobility in Italian Neolithic and Copper Age communities
Source: Sci Rep. 2021 Jan 29;11:2697. doi: 10.1038/s41598-021-81656-z (PMC7846752; doi:10.1038/s41598-021-81656-z)
Supplement: Supplementary file 1 — Supplementary Information 1. [file 41598_2021_81656_MOESM1_ESM.docx]

**Exploring mobility in Italian Neolithic and Copper Age communities.**

De Angelis Flavio°^1*^, Pellegrini Maura°^2,3*^, Martínez-Labarga Cristina ^1^, Anzivino Laura ^1^, Scorrano Gabriele ^1^_§_, Brilli Mauro ^4^, Giustini Francesca ^4^, Angle Micaela^5^, Calattini Mauro^7^, Carboni Giovanni^8^, Catalano Paola^6^, Ceccaroni Emanuela^9^, Cosentino Serena^10^, Di Giannantonio Stefania^6^, Isola Ilaria^11^, Martini Fabio^12^, Pacciani Elsa^13^, Radina Francesca^14^; Rolfo Mario Federico^15^, Silvestrini Mara^16^, Volante Nicoletta^7^, Zanchetta Giovanni^17^, Sarti Lucia^7^ and Rickards Olga^1^.

° Both authors contributed equally to this manuscript

^1^ Centre of Molecular Anthropology for Ancient DNA Studies; University of Rome “Tor Vergata”. Via della Ricerca Scientifica 1, 00133, Rome, Italy. [flavio.de.angelis@uniroma2.it](mailto:flavio.de.angelis@uniroma2.it); martine@uniroma2.it; [laura.anzivino@yahoo](mailto:laura.anzivino@yahoo).it; rickards@uniroma2.it

^2^ Thermo Fisher Scientific. Strada Rivoltana 4, 20053 Rodano (MI), Italy. [maura.pellegrini@thermofisher.com](mailto:maura.pellegrini@thermofisher.com)

^3^ Research Laboratory for Archaeology and the History of Art. Dyson Perrins Building, South Parks Road, Oxford OX13QY. maurapellegrini@tin.it

^4^ Istituto di Geologia Ambientale e Geoingegneria (IGAG), CNR, Area della Ricerca di Roma RM1. Via Salaria km 29,300, 00016 Monterotondo Stazione, Rome, Italy. [francesca.giustini@igag](mailto:francesca.giustini@igag).cnr.it; [mauro.brilli@igag](mailto:mauro.brilli@igag).cnr.it

^5^ Istituto Autonomo Villa Adriana e Villa d’Este. Piazza Trento, 5, 00019 Tivoli (RM), Italy [micaela.angle@beniculturali](mailto:micaela.angle@beniculturali).it

^6^ Soprintendenza Speciale Archeologia, Belle Arti e Paesaggio di Roma. Piazza dei Cinquecento, 67, 00185, Rome, Italy. [paola.catalano@beniculturali](mailto:paola.catalano@beniculturali).it; stefidgs@libero.it

^7^ Dipartimento di Scienze storiche e dei Beni culturali, Siena University. Via Roma 56, 53100 Siena, Italy. [mauro.calattini@unisi](mailto:mauro.calattini@unisi).it; [nicoletta.volante@unisi](mailto:nicoletta.volante@unisi).it; martinisarti@alice.it

^8^ Dipartimento di Scienze dell’Antichità, Sapienza University of Rome. P.le A. Moro, 5 – 00185 Rome, Italy. [giovanni.carboni@uniroma](mailto:giovanni.carboni@uniroma)1.it

^9^ Soprintendenza Speciale Archeologia, Belle Arti e Paesaggio dell’Abruzzo. Via degli Agostiniani 14, 66100, Chieti, Italy. [emanuela.ceccaroni@beniculturali](mailto:emanuela.ceccaroni@beniculturali).it.

^10^ Collaborator of Soprintendenza Speciale Archeologia, Belle Arti e Paesaggio dell’Abruzzo. Via degli Agostiniani 14, 66100, Chieti, Italy. [serena.cosentino@yahoo](mailto:serena.cosentino@yahoo).it

^11^Istituto Nazionale di Geofisica e Vulcanologia, Sezione di Pisa, Via Cesare Battisti, 53, 56125 Pisa Italy. [Ilaria.isola@ingv.it](mailto:Ilaria.isola@ingv.it)

^12^ Dipartimento di Storia, Archeologia, Geografia, Arte e Spettacolo-Archeologia preistorica Unit, Florence University. Via S. Egidio 21 50122 Florence, Italy. fabio.martini@unifi.it

^13^ Former Soprintendenza Archeologia, Belle Arti e Paesaggio di Firenze, Pistoia e Prato. Florence, Italy. elsani@dada.it

^14^ Soprintendenza Archeologia Belle Arti e Paesaggio per la città metropolitana di Bari. Via Pier L'Eremita, 25, 70122 Bari, Italy. francesca.radina@beniculturali.it

^15^ Dipartimento di Storia, Patrimonio culturale, Formazione e Società. University of Rome “Tor Vergata”. Via Columbia n. 1 - 00133 Roma. rolfo@uniroma2.it

^16^ Former Soprintendenza per i Beni Archeologici delle Marche. Via Birarelli 18, 60100 Ancona, Italy. silvestrinimara@libero.it

^17^Dipartimento di Scienze della Terra and Centro Interdipartimentale per lo studio dell’Impatto dei Cambiamenti Climatici (CIRSEC), University of Pisa, Via S. Maria 53 56126 Pisa, Italy

_§_ Current affiliation: Lundbeck Foundation GeoGenetics Centre, GLOBE Institute, University of Copenhagen, Øster Voldgade 5-7, 1350K Copenhagen, Denmark gabrielescor@gmail.com, g.scorrano@sund.ku.dk

**Corresponding authors**: *Flavio De Angelis, Centre of Molecular Anthropology for Ancient DNA Studies; University of Rome “Tor Vergata”, Via della Ricerca Scientifica 1, 00133, Rome, Italy. [flavio.de.angelis@uniroma2.it](mailto:flavio.de.angelis@uniroma2.it)

*Maura Pellegrini, Research Laboratory for Archaeology and the History of Art. Dyson Perrins Building, South Parks Road, Oxford OX13QY. maurapellegrini@tin.it

**SUPPLEMENTARY DESCRIPTION FOR ARCHEOLOGICAL CONTEXTUALIZATION**

**Neolithic sites**

The Neolithic Apulian necropolis of Galliano-Palagiano is located on a plain not far away from the coast, just north-west of Taranto. It consists of ten funerary structures, one simple pit, and nine artificial caves, which could have been routinely reused by removing the previous skeletal remains in the back of the cave. Bodies were often found in a curled-up position on their right side with a south-north orientation in the center of the cave, occasionally placed on flat pebbles or surrounded by a rocky fence. Pottery items were not widespread and seem to have been almost exclusively found in graves with skeletons osteologically identified as female [1]. Several women seem to have been significant in the community as indicated by the presence of remarkably refined pottery goods such as the Serra d’Alto typological frame, which can contain ceremonial compounds.

Mora Cavorso cave is located in the Simbruini mountains in south-east Latium, close to the upper Aniene river. The cave is characterized by a wide entrance that suddenly narrows to a tunnel leading to multiple cellars where 28 people along with several domesticated animals dating back to the early Neolithic were found. The recovery of pottery items, together with polished stone axes and some painted vascular assemblages, resembles typological associations with Abruzzi cultural horizons, suggesting trans-Apennine contacts among Central Italy’s first farmers [2] [3] [4].

**Copper Age sites**

The Eneolithic site of Grotta Nisco is one of the several karst caves scattered in the Apulian upland area Alta Murgia at about 380 meters above sea level. The site has been intensively used for funerary purposes and a considerable number of lithic, copper, and pottery items have been recovered [5] and related to the Laterza cultural context, though some distinctive and ancient characteristics were retained. Human skeletal remains found there could belong to 19 individuals. Evaluation of their musculoskeletal stress markers, along with the large set of faunal specimens, suggests the community could have been extensively engaged in livestock breeding and hunting rather than farming [6].

The burial ground of Celano Pratovecchio was recently identified in Abruzzi, close to the shore of the ancient lacustrine area of the Fucino basin [7]. The ceramics found there do not support noticeable trade routes developed by people in Celano Pratovecchio, where the typological associations of pottery could be outlined with the close settlements of Cerchio Capazzano [8]. The recovery of lithic arrowheads along with items related to spinning fibers into textiles, like whorls, identifies the subsistence strategy of people buried in this site as deriving from hunting and breeding.

One of the best archeologically characterized Copper Age burial grounds is Fontenoce di Recanati, lying in the Potenza river plain in the Marche region. This area has been densely populated since the Neolithic and its morphology affected the subsistence strategy of the local communities. The upstream hilly area was specifically devoted to seasonal breeding activities, while the downstream plain was the site of intensive farming and stable settlements. One of the dwellings, on the left shore of Potenza river, was close to Fontenoce di Recanati burial ground. Archeological evidence suggests that it could have originally hosted various individuals from scattered nearby villages, whose funerary rituals were common and tightly linked to the development of artificial caves (the “a grotticella” tombs). The burials show significant typological and cultural affinities with the Aegeo-Anatolian area and can be cross-referenced with the coeval Tyrrhenian Rinaldone cultural context, spread throughout the southern Tuscany area, even though some cultural affinities could also be identified with Apulian necropolises [9]. These cultural similarities are seen in the structural development of the tombs as well as in a shared pottery style, which might be ascribed to contacts between communities for trading and cultural exchanges.

Buca di Spaccasasso is located in the northern foothills of the Uccellina Mountains, at the southern fringe of the Grosseto plain (southern Tuscany). The funerary area is set 120 m above sea level, in ​​an abandoned Neolithic cinnabar mine. Mining created a small plateau bordered by a vertical rock face and a deep well at the base of the vertical rock wall. The Copper Age funerary evidence consists of a collective burial with secondary deposits arranged in different settings including the mining well, a rectangular enclosure built at the base of the rock face, and other areas on the plateau. Human skeletal remains were arranged with artifacts (pottery, arrowheads, and beads) interpreted as offerings or grave goods in each of the site’s areas, following different steps of the funerary ritual. The rectangular enclosure contained a jumble of highly fragmented and sometimes burned human remains (belonging to at least 61 individuals), hundreds of potsherds and a smaller percentage of lithic tools and beads that could indicate an “ossuary” or container for the ordinary management of the funerary area. The pottery from the rectangular enclosure appears typologically comparable to central and southern Italian cultural areas [10] [11]. The nearby dwellings have not been identified, making it impossible to investigate if people buried at Buca di Spaccasasso came from the coastal hillside of the ancient lagoon of Grosseto or more distant places. All fifty samples selected for analysis in this research come from the ossuary enclosure.

Archeological investigations carried out in the suburbs of Rome during the past few years have discovered several burial grounds and large settlements traceable to the Copper Age [12]. The investigated sites span over 15 centuries and were located within the hydrographic network of the left tributaries of the Aniene river. The richness of the archeological data related to funeral practices is only partially supported by anthropological evaluation due to the poor preservation of the skeletal remains.

The structures and the typological elements at Casetta Mistici suggest an affinity with the Rinaldone culture complex, a Copper Age cultural landscape characterizing the southern Tuscany and the northern edge of Latium. Nonetheless, residential levels referable to Gaudo cultural context are meaningful since they witnessed permanent settlements belonging to this culture outside the Campania region [13] [14] [15].

The necropolis of Torre della Chiesaccia is located close to the settlement of Tor Pagnotta, south of Rome. It belonged to an elitarian community, possibly living close to the burial ground. Material culture elements belonged to the southern Gaudo cultural context: vessels, weapons, and metal artifacts are stylistically similar to Campanian goods. A tomb within this necropolis stands out due to certain peculiarities: cinnabar traces scattered on the chamber floor, a copper dagger and a flint item found in a pit, and other artifacts and weapons placed as grave goods. A rare Campanian-style “nutshell” vessel can be linked to the southern Piana di Sorrento necropolis [12] [14], suggesting contacts among communities living in these areas.

The settlement of Osteria del Curato-via Cinquefrondi is located in the eastern suburbs of Rome, close to other coeval settlements such as Piscina di Torre Spaccata and Quadrato di Torre Spaccata [16], and along one of the main ancient tributaries of the Aniene river, the Gregna channel. The northwestern fringe of the settlement was contiguous with a necropolis where several cultural contexts (Rinaldone, Ortucchio, and Laterza) overlapped [14]. The meaningful topographic distribution of the tombs is remarkable: some graves were within the boundaries of the settlement and generally lacked goods, whereas all the burials in the necropolis shared a single ritually fragmented vessel, and in some cases, flint arrowheads. The differing value of the goods was putatively hypothesized as related to the different origins of the people buried inside the tombs.

Pantano Borghese is located south of Rome, in a swampy plain where several small Copper Age cemetery and dwellings were identified. Heterogeneous funerary practices were identified in Pantano Borghese. Pottery artifacts link people buried in Pantano Borghese to the neighboring Osteria del Curato-via Cinquefrondi community, but also to the southern Gaudo communities, facilitated by the Sacco-Liri valley that could have been represented a preferential route between south Rome area and Campania [17] [18].

Supplementary Figure S1


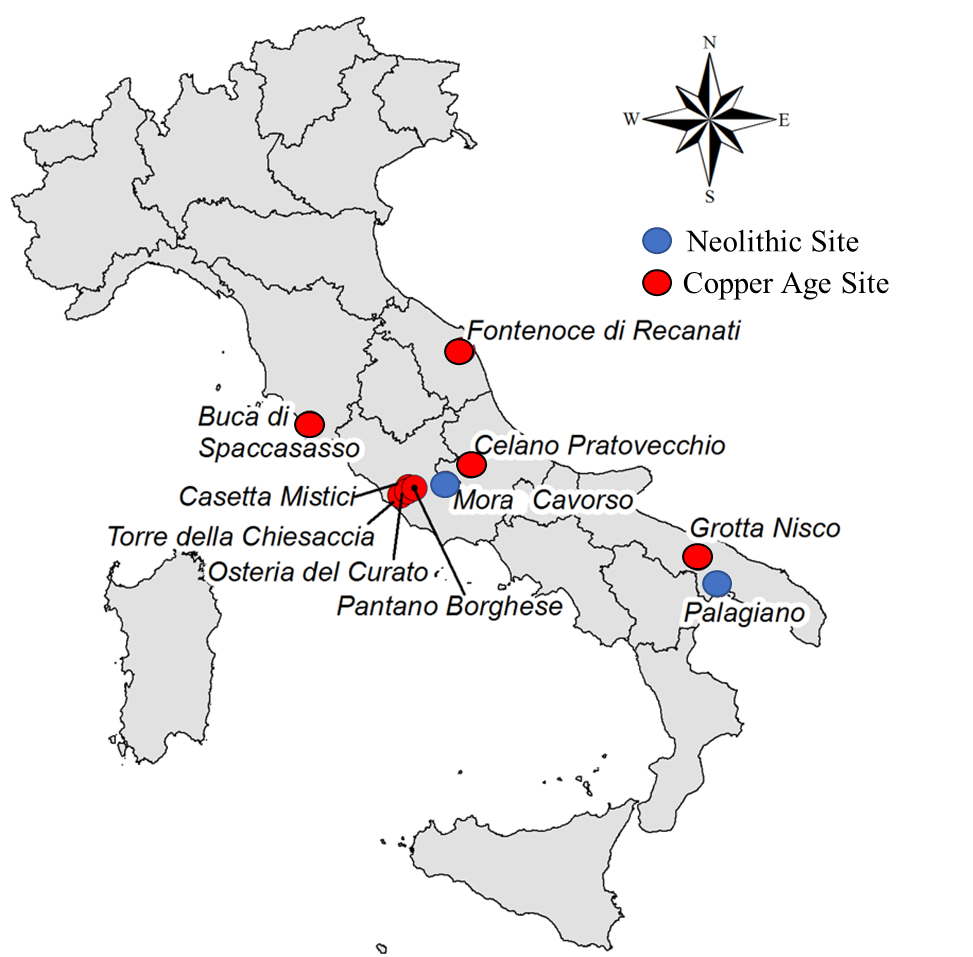


**FIGURE CAPTION**

Supplementary Figure S1: Geographical locations of the sites. Italian administrative boundaries from ISTAT geoportal (available at https://www.istat.it/it/archivio/222527)

**SUPPLEMENTARY TABLE S1**

| Site | n | Mean δ^18^Oca  ‰ V-SMOW | Standard Deviation δ^18^Oca  ‰ V-SMOW | Mean δ^18^Odw  after [53] in main text | Standard Deviation δ^18^Odw  after [53] in main text |
| --- | --- | --- | --- | --- | --- |
| Buca di Spaccasasso | 50 | 26.8 | 0.6 | -6.0 | 0.9 |
| Fontenoce di Recanati | 18 | 24.4 | 1.2 | -9.9 | 1.9 |
| Celano Pratovecchio | 3 | 24.5 | 0.3 | -9.7 | 0.4 |
| Eneolithic Rome | 30 | 26.2 | 0.7 | -7.0 | 1.1 |
| Grotta Nisco | 7 | 26.9 | 0.5 | -5.9 | 0.8 |
| Galliano Palagiano | 9 | 25.4 | 0.7 | -8.3 | 1.1 |
| Mora Cavorso | 9 | 25.9 | 0.5 | -7.5 | 0.9 |

Supplementary Table S1: Converted values according to [53] in main text.

**SUPPLEMENTARY TABLE S2**

| Site | Tomb | Lab Code | Tooth | Age | Sex |
| --- | --- | --- | --- | --- | --- |
| Fontenoce Recanati | 2 | FN 2 | dm | 1-3 | NA |
| Fontenoce Recanati | 3 | FN 3.1 | M^2^R | 10-11 | NA |
| Fontenoce Recanati | 4 | FN 4.1 | dm^1^R | 4-5 | NA |
| Fontenoce Recanati | 5 | FN 5 | M^1^L | 12-14 | NA |
| Fontenoce Recanati | 6 | FN 6.1 | dc | 2-3 | NA |
| Fontenoce Recanati | 6 | FN 6.2 | P | 12-13 | NA |
| Fontenoce Recanati | 7 | FN 7 | dm^1^L | 1-3 | NA |
| Fontenoce Recanati | 9 | FN 9.1 | M^2^L | 18-19 | M |
| Fontenoce Recanati | 9 | FN 9.2 | M^2^L | 13-15 | NA |
| Fontenoce Recanati | 11 | FN 11.1 | P^2^L | adult | F |
| Fontenoce Recanati | 11 | FN 11.2 | dm | 3-5 | NA |
| Fontenoce Recanati | 12 | FN 12.2 | M^2^R | adult | F |
| Fontenoce Recanati | 15 | FN 15 | M^3^L | adult | F |
| Fontenoce Recanati | 16 | FN 16 | M^3^L | adult | M |
| Fontenoce Recanati | 19 | FN 19.1 | M^1^ | adult | M |
| Fontenoce Recanati | 19 | FN 19.2 | dm^2^R | 2-4 | NA |
| Fontenoce Recanati | 20 | FN 20.1 | M^3^ | adult | F |
| Fontenoce Recanati | 21 | FN 21 | M^2^R | adult | F |
| Grotta Nisco | Area2 | GN SCTL | M_1_R | adult | NA |
| Grotta Nisco | Area2 | GN SC2 | M_1_R | adult | NA |
| Grotta Nisco | Area2 | GN SCTD | M^1^R | adult | NA |
| Grotta Nisco | Area2 | GN 1 | M^2^L | adult | NA |
| Grotta Nisco | Area2 | GN 2 | M^2^L | adult | NA |
| Grotta Nisco | Area2 | GN 4 | M^1^R | adult | NA |
| Grotta Nisco | Area2 | GN 8 | M^2^L | adult | NA |
| Celano | 249 | CEL249 | dc^1^L | NA | NA |
| Celano | 250 | CEL250 | M_3_R | adult | M |
| Celano | 253 | CEL253 | M^2^L | adult | M |
| Pantano Borghese | 4 | PB 4 | M^1^R | 5-7 | NA |
| Pantano Borghese | 12 | PB 12 | I^1^L | 25-35 | F |
| Pantano Borghese | 18 | PB 18 | M_2_ (fragment) | 50-60 | M |
| Buca di Spaccasasso | SPS04 E0 US5 Ty6CCCXXXIV | SPS1 | M_2_R | adult | NA |
| Buca di Spaccasasso | SPS04 E1 US100 TyT5CCCXLI | SPS2 | M_2_R | adult | NA |
| Buca di Spaccasasso | SPS08 US45 CO III 15517a | SPS3 | M_2_R | adult | NA |
| Buca di Spaccasasso | SPS08 US35 D1 IV 15140c | SPS4 | M_2_R | adult | NA |
| Buca di Spaccasasso | SPS08 US51 DO IV 15557b | SPS5 | M_2_R | adult | NA |
| Buca di Spaccasasso | SPS2011 US73 base 18001a | SPS6 | M_2_R | adult | NA |
| Buca di Spaccasasso | SPS04 E1 US100 Ty tsCCCXL | SPS7 | M_2_R | adult | NA |
| Buca di Spaccasasso | 13910 | SPS_8p | M_2_R | adult | NA |
| Buca di Spaccasasso | 140070U | SPS9p | M_2_R | adult | NA |
| Buca di Spaccasasso | 17538 | SPS10p | M_2_R | adult | NA |
| Buca di Spaccasasso | 580 18106 | SPS11p | M_2_R | adult | NA |
| Buca di Spaccasasso | 13971 | SPS12p | M_2_R | adult | NA |
| Buca di Spaccasasso | 13540U | SPS13p | M_2_R | adult | NA |
| Buca di Spaccasasso | 141760U | SPS14 | M_2_R | adult | NA |
| Buca di Spaccasasso | 14359 | SPS15p | M_2_R | adult | NA |
| Buca di Spaccasasso | 15822 | SPS16 | M_2_R | adult | NA |
| Buca di Spaccasasso | 17828 | SPS17p | M_2_R | adult | NA |
| Buca di Spaccasasso | 13796b | SPS18p | M_2_R | adult | NA |
| Buca di Spaccasasso | 14177a | SPS_19p | M_2_R | adult | NA |
| Buca di Spaccasasso | 15769 | SPS_20p | M_2_R | adult | NA |
| Buca di Spaccasasso | 15525 | SPS_21p | M_2_R | adult | NA |
| Buca di Spaccasasso | 14280 | SPS_22p | M_2_R | adult | NA |
| Buca di Spaccasasso | 14238 | SPS_23p | M_2_R | adult | NA |
| Buca di Spaccasasso | 14411a | SPS_24p | M_2_R | adult | NA |
| Buca di Spaccasasso | US 16 | SPS_25p | M_2_R | adult | NA |
| Buca di Spaccasasso | US 90 | SPS_26p | M_2_R | adult | NA |
| Buca di Spaccasasso | 15739 | SPS_27p | M_2_R | adult | NA |
| Buca di Spaccasasso | 17725 | SPS_28p | M_2_R | adult | NA |
| Buca di Spaccasasso | 14179a | SPS_29p | M_2_R | adult | NA |
| Buca di Spaccasasso | 15712 | SPS_30p | M_2_R | adult | NA |
| Buca di Spaccasasso | 15774 | SPS_31p | M_2_R | adult | NA |
| Buca di Spaccasasso | US2tyt4 | SPS_32p | M_2_R | adult | NA |
| Buca di Spaccasasso | 13431 | SPS_33p | M_2_R | adult | NA |
| Buca di Spaccasasso | 18456 | SPS_34p | M_2_R | adult | NA |
| Buca di Spaccasasso | 16484 | SPS_35p | M_2_R | adult | NA |
| Buca di Spaccasasso | 17167 | SPS_36p | M_2_R | adult | NA |
| Buca di Spaccasasso | 14129 | SPS_37p | M_2_R | adult | NA |
| Buca di Spaccasasso | US 100 TyT5 | SPS38 | M_2_R | adult | NA |
| Buca di Spaccasasso | 14603 | SPS_39p | M_2_R | adult | NA |
| Buca di Spaccasasso | 15507 | SPS_40p | M_2_R | adult | NA |
| Buca di Spaccasasso | 14003 | SPS_41p | M_2_R | adult | NA |
| Buca di Spaccasasso | 17905 | SPS_42p | M_2_R | adult | NA |
| Buca di Spaccasasso | 14014 | SPS_43p | M_2_R | adult | NA |
| Buca di Spaccasasso | 14364 | SPS_44p | M_2_R | adult | NA |
| Buca di Spaccasasso | 18070 | SPS_45p | M_2_R | adult | NA |
| Buca di Spaccasasso | US 45 CO II | SPS_46p | M_2_R | adult | NA |
| Buca di Spaccasasso | US 27 147809 | SPS_47p | M_2_R | adult | NA |
| Buca di Spaccasasso | 17725 B | SPS_48p | M_2_R | adult | NA |
| Buca di Spaccasasso | 14324 | SPS_49p | M_2_R | adult | NA |
| Buca di Spaccasasso | US 76 CO III | SPS_50p | M_2_R | adult | NA |
| Torre della Chiesaccia | 2 US4 VI t | TC2.A | M^1^R | 20-30 | NA |
| Torre della Chiesaccia | 2 US4 VI t | TC2.B | M_2_R | 6-12 | NA |
| Torre della Chiesaccia | 3 | TC3 | dm_1_L | child | NA |
| Torre della Chiesaccia | 4 US12 VII t | TC4.B | M_1_R | 16-21 | NA |
| Osteria del Curato Via Cinquefrondi | 1 | CF1 | M^2^R | 6-8 | NA |
| Osteria del Curato Via Cinquefrondi | 2 | CF2.2 | M_1_L | 20-25 | NA |
| Osteria del Curato Via Cinquefrondi | 7 | CF7.2 | M_2_R | 18-20 | NA |
| Osteria del Curato Via Cinquefrondi | 7 | CF7.3 | M_1_L | 7-11 | NA |
| Osteria del Curato Via Cinquefrondi | 9 | CF9 | M_1_L | 20-25 | M |
| Osteria del Curato Via Cinquefrondi | 15 | CF15.2 | M_2_R | 18-20 | NA |
| Osteria del Curato Via Cinquefrondi | 17 | CF17 | M_2_L | 30-40 | F |
| Osteria del Curato Via Cinquefrondi | 18 areaG US2383 | CF18 | M_1_L | 18-20 | NA |
| Osteria del Curato Via Cinquefrondi | 19 | CF19 | M^2^L | >50 | M |
| Osteria del Curato Via Cinquefrondi | 21 | CF21 | M^1^L | >18 | NA |
| Osteria del Curato Via Cinquefrondi | 22 | CF22 | dm_1_R | 2-4 | NA |
| Osteria del Curato Via Cinquefrondi | 23 | CF23 | M^2^R | 30-40 | F |
| Osteria del Curato Via Cinquefrondi | 24 | CF24 | M_1_L | 35-40 | M |
| Osteria del Curato Via Cinquefrondi | 26 | CF26 | M_2_R | 18-20 | F |
| Osteria del Curato Via Cinquefrondi | 27 | CF27 | M^1^L | 20-30 | F |
| Osteria del Curato Via Cinquefrondi | 30 areaG US2423 | CF30 | dm_1_L | 2-4 | NA |
| Palagiano | 1 | PAL1 | M^3^L | adult | NA |
| Palagiano | 3.1 | PAL4 | P^1^ | 9-13 | NA |
| Palagiano | 3.2 | PAL7 | P^1^ | 9-14 | NA |
| Palagiano | 4 | PAL10 | M^1^R | adult | NA |
| Palagiano | 5 | PAL13 | M^3^R | adult | NA |
| Palagiano | 6 | PAL16 | M^3^R | adult | NA |
| Palagiano | 10.1 | PAL19 | M^3^ | adult | NA |
| Palagiano | 11.1 | PAL24 | M_2_ | adult | NA |
| Palagiano | 12 | PAL27 | C^R^ | adult | NA |
| Casetta Mistici | T1NEC | T1NEC | I_2_R | 1-2 | NA |
| Casetta Mistici | T3AM | T3AM | M^1^L | 30-40 | F |
| Casetta Mistici | T8NEC | T8NEC | M_1_R | 30-40 | M |
| Casetta Mistici | T10NEC | T10NEC | M_1_R | 20-30 | NA |
| Casetta Mistici | T6BNEC | T6BNEC | C^R^ | 13-19 | NA |
| Casetta Mistici | T6ANEC | T6ANEC | M_1_R | 13-19 | NA |
| Casetta Mistici | T4A | T4A | M_1_R | 25-35 | F |
| Mora Cavorso | 12C+13+12/23 | 12/23 | M_2_R | 35-40 | NA |
| Mora Cavorso | 102 | 102 | M_2_R | 20-25 | F |
| Mora Cavorso | 65B | 65B | M_2_R | 20-25 | M |
| Mora Cavorso | 39VB | 39VB | M_2_R | 17-20 | M |
| Mora Cavorso | 11* | 11* | M_2_R | 17-25 | NA |
| Mora Cavorso | 11*B | 11*B | M_2_R | 6-8 | NA |
| Mora Cavorso | 11? | 11? | M_2_R | adult | NA |
| Mora Cavorso | 12 | 12 | M_2_R | 20-25 | NA |
| Mora Cavorso | 13* | 13* | M_2_R | 17-25 | NA |

Supplementary Table S2: Sex and age at death scored for the sampled individuals. Capital letters indicates Adult dentition; lowercase letters indicate deciduous dentition. Numbers refer to teeth sequence. L: left; R: right. I: Incisor; C: Canine, P: Premolar; M: Molar; F: Female, M: Male, NA: Not available. Age is expressed in years.

**SUPPLEMENTARY TABLE S3**

| Site | Code | δ^13^C_ca_ | δ^18^O_ca_ V-SMOW | δ^18^O_ca_ V-PDB |
| --- | --- | --- | --- | --- |
| Fontenoce di Recanati | FN 2 | -11.0 | 24.1 | -6.6 |
| Fontenoce di Recanati | FN 3.1 | -9.7 | 22.9 | -7.7 |
| Fontenoce di Recanati | FN 4.1 | -11.0 | 23.9 | -6.8 |
| Fontenoce di Recanati | FN 5 | -11.9 | 24.9 | -5.8 |
| Fontenoce di Recanati | FN 6.1 | -9.5 | 21.6 | -9.0 |
| Fontenoce di Recanati | FN 6.2 | -11.9 | 24.8 | -5.9 |
| Fontenoce di Recanati | FN 7 | -12.2 | 24.3 | -6.4 |
| Fontenoce di Recanati | FN 9.1 | -10.7 | 23.8 | -6.9 |
| Fontenoce di Recanati | FN 9.2 | -13.4 | 24.6 | -6.1 |
| Fontenoce di Recanati | FN 11.1 | -12.6 | 26.3 | -4.5 |
| Fontenoce di Recanati | FN 11.2 | -10.9 | 24.4 | -6.4 |
| Fontenoce di Recanati | FN 12.2 | -13.2 | 25.5 | -5.2 |
| Fontenoce di Recanati | FN 15 | -12.7 | 25.3 | -5.4 |
| Fontenoce di Recanati | FN 16 | -12.7 | 24.5 | -6.2 |
| Fontenoce di Recanati | FN 19.1 | -10.8 | 22.2 | -8.4 |
| Fontenoce di Recanati | FN 19.2 | -12.4 | 24.9 | -5.8 |
| Fontenoce di Recanati | FN 20.1 | -12.0 | 26.0 | -4.7 |
| Fontenoce di Recanati | FN 21 | -10.8 | 24.2 | -6.5 |
| Grotta Nisco | GN SC2 | -11.8 | 26.8 | -4.0 |
| Grotta Nisco | GN SCTL | -12.3 | 26.9 | -3.9 |
| Grotta Nisco | GN SCTD | -12.2 | 26.5 | -4.3 |
| Grotta Nisco | GN 1 | -13.1 | 27.7 | -3.1 |
| Grotta Nisco | GN 2 | -13.1 | 26.9 | -3.9 |
| Grotta Nisco | GN 4 | -12.6 | 26.0 | -4.8 |
| Grotta Nisco | GN 8 | -12.2 | 27.2 | -3.6 |
| Celano Pratovecchio | CEL249 | -11.1 | 24.8 | -6.3 |
| Celano Pratovecchio | CEL250 | -12.4 | 24.3 | -5.9 |
| Celano Pratovecchio | CEL253 | -13.6 | 24.4 | -6.4 |
| Pantano Borghese | PB 4 | -11.5 | 26.3 | -4.5 |
| Pantano Borghese | PB 12 | -11.9 | 26.2 | -4.6 |
| Pantano Borghese | PB 18 | -13.4 | 26.2 | -4.6 |
| Buca di Spaccasasso | SPS1 | -14.8 | 27.3 | -3.5 |
| Buca di Spaccasasso | SPS2 | -14.9 | 27.5 | -3.3 |
| Buca di Spaccasasso | SPS3 | -12.7 | 26.6 | -4.2 |
| Buca di Spaccasasso | SPS4 | -12.5 | 27.9 | -3.0 |
| Buca di Spaccasasso | SPS5 | -13.6 | 27.4 | -3.4 |
| Buca di Spaccasasso | SPS6 | -12.6 | 26.9 | -3.9 |
| Buca di Spaccasasso | SPS7 | -13.3 | 26.6 | -4.2 |
| Buca di Spaccasasso | SPS_8p | -13.0 | 26.5 | -4.3 |
| Buca di Spaccasasso | SPS9p | -10.9 | 26.5 | -4.3 |
| Buca di Spaccasasso | SPS10p | -12.6 | 26.8 | -4.0 |
| Buca di Spaccasasso | SPS11p | -13.5 | 25.6 | -5.2 |
| Buca di Spaccasasso | SPS12p | -13.6 | 27.6 | -3.2 |
| Buca di Spaccasasso | SPS13p | -14.1 | 27.1 | -3.7 |
| Buca di Spaccasasso | SPS14 | -9.9 | 26.3 | -4.5 |
| Buca di Spaccasasso | SPS15p | -13.7 | 26.8 | -4.0 |
| Buca di Spaccasasso | SPS16 | -11.1 | 26.9 | -3.9 |
| Buca di Spaccasasso | SPS17p | -13.6 | 27.0 | -3.8 |
| Buca di Spaccasasso | SPS18p | -14.0 | 27.0 | -3.7 |
| Buca di Spaccasasso | SPS_19p | -13.6 | 27.2 | -3.6 |
| Buca di Spaccasasso | SPS_20p | -13.0 | 26.9 | -3.9 |
| Buca di Spaccasasso | SPS_21p | -13.9 | 27.1 | -3.7 |
| Buca di Spaccasasso | SPS_22p | -14.4 | 26.4 | -4.4 |
| Buca di Spaccasasso | SPS_23p | -14.2 | 24.8 | -6.0 |
| Buca di Spaccasasso | SPS_24p | -12.0 | 26.7 | -4.1 |
| Buca di Spaccasasso | SPS_25p | -13.2 | 25.5 | -5.3 |
| Buca di Spaccasasso | SPS_26p | -12.5 | 26.5 | -4.3 |
| Buca di Spaccasasso | SPS_27p | -12.9 | 26.8 | -4.0 |
| Buca di Spaccasasso | SPS_28p | -13.1 | 26.7 | -4.1 |
| Buca di Spaccasasso | SPS_29p | -13.5 | 27.2 | -3.6 |
| Buca di Spaccasasso | SPS_30p | -14.6 | 27.5 | -3.3 |
| Buca di Spaccasasso | SPS_31p | -14.2 | 26.6 | -4.1 |
| Buca di Spaccasasso | SPS_32p | -12.8 | 26.4 | -4.3 |
| Buca di Spaccasasso | SPS_33p | -13.3 | 27.1 | -3.7 |
| Buca di Spaccasasso | SPS_34p | -12.4 | 27.2 | -3.6 |
| Buca di Spaccasasso | SPS_35p | -13.7 | 26.2 | -4.6 |
| Buca di Spaccasasso | SPS_36p | -13.3 | 27.2 | -3.6 |
| Buca di Spaccasasso | SPS_37p | -12.8 | 27.2 | -3.6 |
| Buca di Spaccasasso | SPS38 | -12.1 | 26.4 | -4.3 |
| Buca di Spaccasasso | SPS_39p | -12.6 | 25.9 | -4.8 |
| Buca di Spaccasasso | SPS_40p | -13.0 | 26.9 | -3.9 |
| Buca di Spaccasasso | SPS_41p | -12.8 | 26.1 | -4.6 |
| Buca di Spaccasasso | SPS_42p | -12.8 | 26.2 | -4.5 |
| Buca di Spaccasasso | SPS_43p | -13.7 | 27.4 | -3.4 |
| Buca di Spaccasasso | SPS_44p | -13.6 | 28.1 | -2.7 |
| Buca di Spaccasasso | SPS_45p | -13.6 | 26.5 | -4.3 |
| Buca di Spaccasasso | SPS_46p | -12.3 | 27.0 | -3.8 |
| Buca di Spaccasasso | SPS_47p | -13.1 | 26.7 | -4.1 |
| Buca di Spaccasasso | SPS_48p | -12.9 | 26.7 | -4.1 |
| Buca di Spaccasasso | SPS_49p | -14.0 | 26.8 | -4.0 |
| Buca di Spaccasasso | SPS_50p | -13.0 | 27.0 | -3.8 |
| Torre della Chiesaccia | TC2.A | -13.9 | 26.9 | -3.9 |
| Torre della Chiesaccia | TC2.B | -14.2 | 26.1 | -4.7 |
| Torre della Chiesaccia | TC3 | -12.7 | 26.5 | -4.3 |
| Torre della Chiesaccia | TC4.B | -13.7 | 26.1 | -4.7 |
| Osteria del Curato-Via Cinquefrondi | CF1 | -13.1 | 26.3 | -4.4 |
| Osteria del Curato-Via Cinquefrondi | CF2.2 | -13.1 | 23.8 | -6.9 |
| Osteria del Curato-Via Cinquefrondi | CF7.2 | -12.5 | 26.2 | -4.6 |
| Osteria del Curato-Via Cinquefrondi | CF7.3 | -14.1 | 25.0 | -5.7 |
| Osteria del Curato-Via Cinquefrondi | CF9 | -13.7 | 26.2 | -4.6 |
| Osteria del Curato-Via Cinquefrondi | CF15.2 | -13.8 | 25.9 | -4.9 |
| Osteria del Curato-Via Cinquefrondi | CF17 | -13.8 | 27.1 | -3.7 |
| Osteria del Curato-Via Cinquefrondi | CF18 | -13.5 | 25.9 | -4.8 |
| Osteria del Curato-Via Cinquefrondi | CF19 | -13.6 | 26.3 | -4.5 |
| Osteria del Curato-Via Cinquefrondi | CF21 | -14.2 | 26.0 | -4.7 |
| Osteria del Curato-Via Cinquefrondi | CF22 | -13.7 | 26.6 | -4.2 |
| Osteria del Curato-Via Cinquefrondi | CF23 | -13.9 | 26.1 | -4.7 |
| Osteria del Curato-Via Cinquefrondi | CF24 | -13.6 | 26.9 | -3.8 |
| Osteria del Curato-Via Cinquefrondi | CF26 | -14.1 | 26.3 | -4.5 |
| Osteria del Curato-Via Cinquefrondi | CF27 | -14.2 | 27.6 | -3.2 |
| Osteria del Curato-Via Cinquefrondi | CF30 | -14.0 | 25.8 | -4.9 |
| Galliano Palagiano | PAL1 | -12.5 | 24.8 | -5.9 |
| Galliano Palagiano | PAL4 | -11.5 | 24.6 | -6.2 |
| Galliano Palagiano | PAL7 | -12.4 | 26.1 | -4.7 |
| Galliano Palagiano | PAL10 | -11.4 | 26.6 | -4.2 |
| Galliano Palagiano | PAL13 | -12.7 | 25.1 | -5.6 |
| Galliano Palagiano | PAL16 | -12.5 | 25.3 | -5.4 |
| Galliano Palagiano | PAL19 | -12.1 | 25.0 | -5.7 |
| Galliano Palagiano | PAL24 | -12.4 | 25.9 | -4.9 |
| Galliano Palagiano | PAL27 | -11.9 | 25.0 | -5.7 |
| Casetta Mistici | T1NEC | -12.2 | 25.1 | -5.6 |
| Casetta Mistici | T3AM | -13.4 | 25.8 | -4.9 |
| Casetta Mistici | T8NEC | -12.9 | 26.0 | -4.7 |
| Casetta Mistici | T10NEC | -11.5 | 26.0 | -4.8 |
| Casetta Mistici | T6BNEC | -12.3 | 26.3 | -4.5 |
| Casetta Mistici | T6ANEC | -13.2 | 26.1 | -4.6 |
| Casetta Mistici | T4A | -13.1 | 26.9 | -3.9 |
| Mora Cavorso | 11*B | -7.0 | 26.4 | -4.4 |
| Mora Cavorso | 11* | -9.7 | 26.3 | -4.5 |
| Mora Cavorso | 12 | -11.1 | 26.0 | -4.8 |
| Mora Cavorso | 11? | -11.3 | 26.2 | -4.6 |
| Mora Cavorso | 13* | -10.8 | 26.4 | -4.4 |
| Mora Cavorso | dic-23 | -6.5 | 25.3 | -5.5 |
| Mora Cavorso | 39VB | -4.4 | 25.2 | -5.5 |
| Mora Cavorso | 65B | -3.8 | 26.0 | -4.8 |
| Mora Cavorso | 102 | -6.6 | 25.1 | -5.6 |

Supplementary Table S3: Individual isotopic ratios.

**SUPPLEMENTARY TABLE S4**

| Speleothem | Sample | δ ^13^C (VPDB) | δ ^18^O (VPDB) | δ ^18^O (VSMOW) |
| --- | --- | --- | --- | --- |
| Speleothem 1 (MIS 5e) | CAV7-1_1 | -11.13 | -6.03 | 24.69 |
| Speleothem 1 (MIS 5e) | CAV7-1_2 | -11.19 | -6.55 | 24.16 |
| Speleothem 1 (MIS 5e) | CAV7-1_3 | -10.93 | -6.11 | 24.61 |
| Speleothem 1 (MIS 5e) | CAV7-1_4 | -10.99 | -6.34 | 24.37 |
| Speleothem 1 (MIS 5e) | CAV7-1_5 | -11.02 | -6.31 | 24.40 |
| Speleothem 1 (MIS 5e) | CAV7-1_6 | -11.06 | -6.30 | 24.42 |
| Speleothem 1 (MIS 5e) | CAV7-1_7 | -11.09 | -5.95 | 24.78 |
| Speleothem 1 (MIS 5e) | CAV7-1_8 | -11.05 | -5.98 | 24.75 |
| Speleothem 1 (MIS 5e) | CAV7-1_9 | -11.14 | -6.92 | 23.78 |
| Speleothem 1 (MIS 5e) | CAV7-1_10 | -10.95 | -6.28 | 24.44 |
| Speleothem 1 (MIS 5e) | CAV7-1_11 | -10.85 | -6.04 | 24.68 |
| Speleothem 1 (MIS 5e) | CAV7-1_12 | -10.91 | -6.01 | 24.71 |
| Speleothem 1 (MIS 5e) | CAV7-1_13 | -10.92 | -6.09 | 24.63 |
| Speleothem 1 (MIS 5e) | CAV7-1_14 | -10.99 | -6.10 | 24.62 |
| Speleothem 1 (MIS 5e) | CAV7-1_15 | -10.82 | -6.05 | 24.67 |
| Speleothem 1 (MIS 5e) | CAV7-1_16 | -10.89 | -6.19 | 24.52 |
| Speleothem 1 (MIS 5e) | CAV7-1_17 | -10.69 | -5.84 | 24.89 |
| Speleothem 1 (MIS 5e) | CAV7-1_18 | -10.63 | -5.88 | 24.85 |
| Speleothem 1 (MIS 5e) | CAV7-1_19 | -10.66 | -5.96 | 24.77 |
| Speleothem 1 (MIS 5e) | CAV7-1_20 | -10.56 | -5.93 | 24.80 |
| Speleothem 1 (MIS 5e) | CAV7-1_21 | -10.77 | -6.02 | 24.70 |
| Speleothem 1 (MIS 5e) | CAV7-1_22 | -10.56 | -6.17 | 24.55 |
| Speleothem 1 (MIS 5e) | CAV7-1_23 | -10.53 | -6.24 | 24.48 |
| Speleothem 1 (MIS 5e) | CAV7-1_24 | -10.36 | -6.05 | 24.68 |
| Speleothem 1 (MIS 5e) | CAV7-1_25 | -10.45 | -6.21 | 24.51 |
| Speleothem 1 (MIS 5e) | CAV7-1_26 | -10.44 | -6.17 | 24.55 |
| Speleothem 1 (MIS 5e) | CAV7-1_27 | -10.51 | -5.59 | 25.15 |
| Speleothem 1 (MIS 5e) | CAV7-1_28 | -10.46 | -6.02 | 24.70 |
| Speleothem 1 (MIS 5e) | CAV7-1_29 | -10.52 | -5.94 | 24.79 |
| Speleothem 1 (MIS 5e) | CAV7-1_30 | -10.46 | -6.06 | 24.66 |
| Speleothem 1 (MIS 5e) | CAV7-1_31 | -10.64 | -6.04 | 24.68 |
| Speleothem 1 (MIS 5e) | CAV7-1_32 | -10.55 | -6.17 | 24.55 |
| Speleothem 1 (MIS 5e) | CAV7-1_33 | -10.53 | -5.93 | 24.80 |
| Speleothem 1 (MIS 5e) | CAV7-1_34 | -10.59 | -5.85 | 24.88 |
| Speleothem 1 (MIS 5e) | CAV7-1_35 | -10.65 | -5.78 | 24.95 |
| Speleothem 1 (MIS 5e) | CAV7-1_36 | -10.53 | -5.58 | 25.16 |
| Speleothem 1 (MIS 5e) | CAV7-1_37 | -10.56 | -5.93 | 24.80 |
| Speleothem 1 (MIS 5e) | CAV7-1_38 | -10.68 | -5.82 | 24.91 |
| Speleothem 1 (MIS 5e) | CAV7-1_39 | -10.82 | -5.95 | 24.78 |
| Speleothem 1 (MIS 5e) | CAV7-1_40 | -10.89 | -5.88 | 24.85 |
| Speleothem 1 (MIS 5e) | CAV7-1_41 | -10.89 | -5.84 | 24.89 |
| Speleothem 1 (MIS 5e) | CAV7-1_42 | -10.87 | -6.20 | 24.52 |
| Speleothem 1 (MIS 5e) | CAV7-1_43 | -10.84 | -6.38 | 24.33 |
| Speleothem 1 (MIS 5e) | CAV7-1_44 | -10.82 | -6.26 | 24.45 |
| Speleothem 1 (MIS 5e) | CAV7-1_45 | -10.91 | -6.27 | 24.45 |
| Speleothem 1 (MIS 5e) | CAV7-1_46 | -10.85 | -5.96 | 24.77 |
| Speleothem 1 (MIS 5e) | CAV7-1_47 | -10.90 | -5.86 | 24.87 |
| Speleothem 1 (MIS 5e) | CAV7-1_48 | -10.80 | -6.00 | 24.72 |
| Speleothem 1 (MIS 5e) | CAV7-1_49 | -10.98 | -6.13 | 24.59 |
| Speleothem 1 (MIS 5e) | CAV7-1_50 | -10.97 | -6.10 | 24.62 |
| Speleothem 1 (MIS 5e) | CAV7-1_51 | -10.88 | -5.91 | 24.81 |
| Speleothem 1 (MIS 5e) | CAV7-1_52 | -10.94 | -5.89 | 24.84 |
| Speleothem 1 (MIS 5e) | CAV7-1_53 | -10.88 | -6.19 | 24.53 |
| Speleothem 1 (MIS 5e) | CAV7-1_54 | -10.95 | -6.59 | 24.12 |
| Speleothem 1 (MIS 5e) | CAV7-1_55 | -10.89 | -6.49 | 24.22 |
| Speleothem 1 (MIS 5e) | CAV7-1_56 | -10.86 | -6.07 | 24.65 |
| Speleothem 1 (MIS 5e) | CAV7-1_57 | -10.85 | -5.91 | 24.82 |
| Speleothem 1 (MIS 5e) | CAV7-1_58 | -10.73 | -5.90 | 24.83 |
| Speleothem 1 (MIS 5e) | CAV7-1_59 | -10.86 | -5.69 | 25.04 |
| Speleothem 1 (MIS 5e) | CAV7-1_60 | -10.84 | -5.92 | 24.80 |
| Speleothem 1 (MIS 5e) | CAV7-1_61 | -10.74 | -5.97 | 24.75 |
| Speleothem 1 (MIS 5e) | CAV7-1_62 | -10.86 | -5.97 | 24.76 |
| Speleothem 1 (MIS 5e) | CAV7-1_63 | -10.79 | -6.19 | 24.53 |
| Speleothem 1 (MIS 5e) | CAV7-1_64 | -10.77 | -6.27 | 24.45 |
| Speleothem 1 (MIS 5e) | CAV7-1_65 | -10.84 | -6.46 | 24.25 |
| Speleothem 1 (MIS 5e) | CAV7-1_66 | -10.67 | -6.27 | 24.45 |
| Speleothem 1 (MIS 5e) | CAV7-1_67 | -10.88 | -6.24 | 24.48 |
| Speleothem 1 (MIS 5e) | CAV7-1_68 | -10.84 | -6.26 | 24.45 |
| Speleothem 1 (MIS 5e) | CAV7-1_69 | -10.85 | -6.12 | 24.60 |
| Speleothem 1 (MIS 5e) | CAV7-1_70 | -10.78 | -5.95 | 24.78 |
| Speleothem 1 (MIS 5e) | CAV7-1_71 | -10.90 | -6.05 | 24.67 |
| Speleothem 1 (MIS 5e) | CAV7-1_72 | -10.85 | -6.04 | 24.68 |
| Speleothem 1 (MIS 5e) | CAV7-1_73 | -10.89 | -6.07 | 24.65 |
| Speleothem 1 (MIS 5e) | CAV7-1_74 | -10.89 | -5.81 | 24.92 |
| Speleothem 1 (MIS 5e) | CAV7-1_76 | -10.73 | -6.02 | 24.70 |
| Speleothem 1 (MIS 5e) | CAV7-1_77 | -10.78 | -5.87 | 24.86 |
| Speleothem 1 (MIS 5e) | CAV7-1_78 | -10.96 | -5.86 | 24.87 |
| Speleothem 1 (MIS 5e) | CAV7-1_79 | -11.03 | -6.01 | 24.72 |
| Speleothem 1 (MIS 5e) | CAV7-1_80 | -11.15 | -5.79 | 24.94 |
| Speleothem 1 (MIS 5e) | CAV7-1_81 | -11.22 | -6.03 | 24.69 |
| Speleothem 1 (MIS 5e) | CAV7-1_82 | -11.58 | -6.55 | 24.16 |
| Speleothem 1 (MIS 5e) | CAV7-1_83 | -11.39 | -6.20 | 24.52 |
| Speleothem 1 (MIS 5e) | CAV7-1_84 | -11.30 | -5.94 | 24.79 |
| Speleothem 1 (MIS 5e) | CAV7-1_85 | -11.37 | -5.84 | 24.89 |
| Speleothem 1 (MIS 5e) | CAV7-1_86 | -11.28 | -5.96 | 24.76 |
| Speleothem 1 (MIS 5e) | CAV7-1_87 | -11.16 | -5.90 | 24.83 |
| Speleothem 1 (MIS 5e) | CAV7-1_88 | -11.05 | -5.35 | 25.39 |
| Speleothem 1 (MIS 5e) | CAV7-1_89 | -10.98 | -5.60 | 25.14 |
| Speleothem 1 (MIS 5e) | CAV7-1_90 | -11.04 | -5.30 | 25.44 |
| Speleothem 1 (MIS 5e) | CAV7-1_91 | -11.20 | -5.34 | 25.41 |
| Speleothem 1 (MIS 5e) | CAV7-1_92 | -11.35 | -5.46 | 25.28 |
| Speleothem 1 (MIS 5e) | CAV7-1_93 | -11.50 | -5.41 | 25.33 |
| Speleothem 1 (MIS 5e) | CAV7-1_94 | -11.36 | -5.42 | 25.32 |
| Speleothem 1 (MIS 5e) | CAV7-1_95 | -11.47 | -5.65 | 25.09 |
| Speleothem 1 (MIS 5e) | CAV7-1_96 | -11.52 | -6.08 | 24.65 |
| Speleothem 1 (MIS 5e) | CAV7-1_97 | -11.56 | -6.12 | 24.60 |
| Speleothem 1 (MIS 5e) | CAV7-1_98 | -11.42 | -6.11 | 24.61 |
| Speleothem 1 (MIS 5e) | CAV7-1_99 | -11.34 | -6.09 | 24.63 |
| Speleothem 1 (MIS 5e) | CAV7-1_100 | -11.42 | -5.85 | 24.88 |
| Speleothem 1 (MIS 5e) | CAV7-1_101 | -11.46 | -5.58 | 25.16 |
| Speleothem 1 (MIS 5e) | CAV7-1_102 | -11.47 | -6.07 | 24.65 |
| Speleothem 1 (MIS 5e) | CAV7-1_103 | -11.43 | -6.22 | 24.49 |
| Speleothem 1 (MIS 5e) | CAV7-1_104 | -11.48 | -5.54 | 25.20 |
| Speleothem 1 (MIS 5e) | CAV7-1_105 | -11.48 | -5.49 | 25.25 |
| Speleothem 1 (MIS 5e) | CAV7-1_106 | -11.62 | -5.97 | 24.76 |
| Speleothem 1 (MIS 5e) | CAV7-1_107 | -11.70 | -6.10 | 24.62 |
| Speleothem 1 (MIS 5e) | CAV7-1_108 | -11.74 | -5.85 | 24.88 |
| Speleothem 1 (MIS 5e) | CAV7-1_109 | -11.66 | -5.75 | 24.99 |
| Speleothem 1 (MIS 5e) | CAV7-1_110 | -11.51 | -5.89 | 24.84 |
| Speleothem 1 (MIS 5e) | CAV7-1_111 | -11.57 | -6.09 | 24.63 |
| Speleothem 1 (MIS 5e) | CAV7-1_112 | -11.41 | -6.34 | 24.38 |
| Speleothem 1 (MIS 5e) | CAV7-1_113 | -11.54 | -5.95 | 24.77 |
| Speleothem 1 (MIS 5e) | CAV7-1_114 | -11.56 | -5.65 | 25.08 |
| Speleothem 1 (MIS 5e) | CAV7-1_115 | -11.53 | -5.62 | 25.12 |
| Speleothem 1 (MIS 5e) | CAV7-1_116 | -11.67 | -5.89 | 24.84 |
| Speleothem 1 (MIS 5e) | CAV7-1_117 | -11.78 | -6.11 | 24.61 |
| Speleothem 1 (MIS 5e) | CAV7-1_118 | -11.75 | -6.19 | 24.53 |
| Speleothem 1 (MIS 5e) | CAV7-1_119 | -11.71 | -5.46 | 25.28 |
| Speleothem 1 (MIS 5e) | CAV7-1_120 | -11.59 | -5.53 | 25.21 |
| Speleothem 1 (MIS 5e) | CAV7-1_121 | -11.53 | -5.66 | 25.07 |
| Speleothem 1 (MIS 5e) | CAV7-1_122 | -11.66 | -5.91 | 24.81 |
| Speleothem 1 (MIS 5e) | CAV7-1_123 | -11.91 | -5.96 | 24.76 |
| Speleothem 1 (MIS 5e) | CAV7-1_124 | -11.72 | -5.74 | 24.99 |
| Speleothem 1 (MIS 5e) | CAV7-1_125 | -11.65 | -6.02 | 24.71 |
| Speleothem 1 (MIS 5e) | CAV7-1_126 | -11.70 | -5.75 | 24.98 |
| Speleothem 1 (MIS 5e) | CAV7-1_127 | -11.64 | -5.99 | 24.73 |
| Speleothem 1 (MIS 5e) | CAV7-1_128 | -11.81 | -6.22 | 24.50 |
| Speleothem 1 (MIS 5e) | CAV7-1_129 | -12.13 | -6.29 | 24.42 |
| Speleothem 1 (MIS 5e) | CAV7-1_130 | -12.07 | -6.61 | 24.10 |
| Speleothem 1 (MIS 5e) | CAV7-1_131 | -12.00 | -6.77 | 23.93 |
| Speleothem 1 (MIS 5e) | CAV7-1_132 | -11.96 | -6.71 | 23.99 |
| Speleothem 1 (MIS 5e) | CAV7-1_133 | -11.72 | -5.81 | 24.92 |
| Speleothem 1 (MIS 5e) | CAV7-1_134 | -11.70 | -5.91 | 24.82 |
| Speleothem 1 (MIS 5e) | CAV7-1_135 | -11.69 | -6.29 | 24.43 |
| Speleothem 1 (MIS 5e) | CAV7-1_136 | -11.50 | -6.06 | 24.66 |
| Speleothem 1 (MIS 5e) | CAV7-1_137 | -11.37 | -5.95 | 24.77 |
| Speleothem 1 (MIS 5e) | CAV7-1_138 | -11.50 | -6.58 | 24.13 |
| Speleothem 1 (MIS 5e) | CAV7-1_139 | -11.33 | -6.39 | 24.32 |
| Speleothem 1 (MIS 5e) | CAV7-1_140 | -11.27 | -6.12 | 24.60 |
| Speleothem 1 (MIS 5e) | CAV7-1_141 | -11.45 | -5.83 | 24.90 |
| Speleothem 1 (MIS 5e) | CAV7-1_142 | -11.55 | -5.92 | 24.80 |
| Speleothem 1 (MIS 5e) | CAV7-1_143 | -11.57 | -5.94 | 24.78 |
| Speleothem 1 (MIS 5e) | CAV7-1_144 | -11.55 | -6.26 | 24.45 |
| Speleothem 1 (MIS 5e) | CAV7-1_145 | -11.63 | -5.91 | 24.82 |
| Speleothem 1 (MIS 5e) | CAV7-1_146 | -11.62 | -5.66 | 25.07 |
| Speleothem 1 (MIS 5e) | CAV7-1_147 | -11.65 | -5.47 | 25.27 |
| Speleothem 1 (MIS 5e) | CAV7-1_148 | -11.75 | -5.73 | 25.01 |
| Speleothem 1 (MIS 5e) | CAV7-1_149 | -11.46 | -5.97 | 24.76 |
| Speleothem 1 (MIS 5e) | CAV7-1_150 | -11.26 | -6.21 | 24.51 |
| Speleothem 1 (MIS 5e) | CAV7-1_151 | -11.32 | -5.92 | 24.81 |
| Speleothem 1 (MIS 5e) | CAV7-1_152 | -11.39 | -5.74 | 25.00 |
| Speleothem 1 (MIS 5e) | CAV7-1_153 | -11.45 | -6.10 | 24.62 |
| Speleothem 1 (MIS 5e) | CAV7-1_154 | -11.28 | -5.97 | 24.76 |
| Speleothem 1 (MIS 5e) | CAV7-1_155 | -11.38 | -5.68 | 25.05 |
| Speleothem 1 (MIS 5e) | CAV7-1_156 | -11.32 | -5.70 | 25.03 |
| Speleothem 1 (MIS 5e) | CAV7-1_157 | -11.25 | -5.91 | 24.82 |
| Speleothem 1 (MIS 5e) | CAV7-1_158 | -11.35 | -5.93 | 24.80 |
| Speleothem 1 (MIS 5e) | CAV7-1_159 | -11.46 | -5.71 | 25.02 |
| Speleothem 1 (MIS 5e) | CAV7-1_160 | -11.19 | -5.69 | 25.04 |
| Speleothem 1 (MIS 5e) | CAV7-1_161 | -11.40 | -5.58 | 25.16 |
| Speleothem 1 (MIS 5e) | CAV7-1_162 | -11.49 | -5.83 | 24.90 |
| Speleothem 1 (MIS 5e) | CAV7-1_163 | -11.56 | -5.86 | 24.87 |
| Speleothem 1 (MIS 5e) | CAV7-1_164 | -11.62 | -5.62 | 25.12 |
| Speleothem 1 (MIS 5e) | CAV7-1_165 | -11.54 | -5.75 | 24.98 |
| Speleothem 1 (MIS 5e) | CAV7-1_166 | -11.52 | -6.20 | 24.52 |
| Speleothem 1 (MIS 5e) | CAV7-1_167 | -11.63 | -6.26 | 24.46 |
| Speleothem 1 (MIS 5e) | CAV7-1_168 | -11.70 | -6.37 | 24.34 |
| Speleothem 1 (MIS 5e) | CAV7-1_169 | -11.91 | -6.01 | 24.71 |
| Speleothem 1 (MIS 5e) | CAV7-1_170 | -11.78 | -6.16 | 24.56 |
| Speleothem 1 (MIS 5e) | CAV7-1_171 | -11.56 | -6.12 | 24.60 |
| Speleothem 1 (MIS 5e) | CAV7-1_172 | -11.37 | -5.88 | 24.85 |
| Speleothem 1 (MIS 5e) | CAV7-1_173 | -11.36 | -5.96 | 24.77 |
| Speleothem 1 (MIS 5e) | CAV7-1_174 | -11.78 | -6.07 | 24.65 |
| Speleothem 1 (MIS 5e) | CAV7-1_175 | -11.81 | -5.97 | 24.76 |
| Speleothem 1 (MIS 5e) | CAV7-1_176 | -11.81 | -5.59 | 25.15 |
| Speleothem 1 (MIS 5e) | CAV7-1_177 | -11.88 | -5.84 | 24.89 |
| Speleothem 1 (MIS 5e) | CAV7-1_178 | -11.67 | -5.11 | 25.64 |
| Speleothem 1 (MIS 5e) | CAV7-1_179 | -11.67 | -5.55 | 25.19 |
| Speleothem 1 (MIS 5e) | CAV7-1_180 | -11.78 | -5.56 | 25.18 |
| Speleothem 1 (MIS 5e) | CAV7-1_181 | -11.95 | -5.72 | 25.01 |
| Speleothem 1 (MIS 5e) | CAV7-1_182 | -12.08 | -6.11 | 24.61 |
| Speleothem 1 (MIS 5e) | CAV7-1_183 | -12.14 | -6.13 | 24.59 |
| Speleothem 1 (MIS 5e) | CAV7-1_184 | -11.90 | -5.86 | 24.87 |
| Speleothem 1 (MIS 5e) | CAV7-1_185 | -11.85 | -6.06 | 24.66 |
| Speleothem 1 (MIS 5e) | CAV7-1_186 | -11.90 | -5.95 | 24.78 |
| Speleothem 1 (MIS 5e) | CAV7-1_187 | -11.98 | -5.90 | 24.83 |
| Speleothem 1 (MIS 5e) | CAV7-1_188 | -12.07 | -5.95 | 24.78 |
| Speleothem 1 (MIS 5e) | CAV7-1_189 | -11.93 | -5.74 | 24.99 |
| Speleothem 1 (MIS 5e) | CAV7-1_190 | -12.18 | -5.63 | 25.11 |
| Speleothem 1 (MIS 5e) | CAV7-1_191 | -11.96 | -5.91 | 24.82 |
| Speleothem 1 (MIS 5e) | CAV7-1_192 | -11.24 | -3.69 | 27.11 |
| Speleothem 1 (MIS 5e) | CAV7-1_193 | -11.80 | -5.85 | 24.88 |
| Speleothem 1 (MIS 5e) | CAV7-1_194 | -11.87 | -6.07 | 24.65 |
| Speleothem 1 (MIS 5e) | CAV7-1_195 | -11.83 | -5.95 | 24.78 |
| Speleothem 1 (MIS 5e) | CAV7-1_196 | -11.88 | -6.16 | 24.56 |
| Speleothem 1 (MIS 5e) | CAV7-1_197 | -11.92 | -6.48 | 24.23 |
| Speleothem 1 (MIS 5e) | CAV7-1_198 | -11.92 | -6.33 | 24.38 |
| Speleothem 1 (MIS 5e) | CAV7-1_199 | -12.04 | -6.05 | 24.67 |
| Speleothem 1 (MIS 5e) | CAV7-1_200 | -12.01 | -5.81 | 24.92 |
| Speleothem 1 (MIS 5e) | CAV7-1_201 | -12.09 | -6.14 | 24.58 |
| Speleothem 1 (MIS 5e) | CAV7-1_202 | -12.36 | -6.25 | 24.47 |
| Speleothem 1 (MIS 5e) | CAV7-1_203 | -12.39 | -6.18 | 24.54 |
| Speleothem 1 (MIS 5e) | CAV7-1_204 | -12.37 | -5.98 | 24.75 |
| Speleothem 1 (MIS 5e) | CAV7-1_205 | -12.54 | -5.70 | 25.03 |
| Speleothem 1 (MIS 5e) | CAV7-1_206 | -12.44 | -5.96 | 24.77 |
| Speleothem 1 (MIS 5e) | CAV7-1_207 | -12.46 | -5.50 | 25.24 |
| Speleothem 1 (MIS 5e) | CAV7-1_208 | -12.21 | -5.56 | 25.18 |
| Speleothem 1 (MIS 5e) | CAV7-1_209 | -12.28 | -5.86 | 24.87 |
| Speleothem 1 (MIS 5e) | CAV7-1_210 | -12.33 | -6.06 | 24.66 |
| Speleothem 1 (MIS 5e) | CAV7-1_211 | -12.05 | -5.50 | 25.24 |
| Speleothem 1 (MIS 5e) | CAV7-1_212 | -12.09 | -5.44 | 25.30 |
| Speleothem 1 (MIS 5e) | CAV7-1_213 | -11.94 | -5.70 | 25.03 |
| Speleothem 1 (MIS 5e) | CAV7-1_214 | -12.36 | -5.80 | 24.93 |
| Speleothem 1 (MIS 5e) | CAV7-1_215 | -12.39 | -6.23 | 24.49 |
| Speleothem 1 (MIS 5e) | CAV7-1_216 | -12.46 | -6.37 | 24.34 |
| Speleothem 1 (MIS 5e) | CAV7-1_217 | -12.24 | -5.69 | 25.04 |
| Speleothem 1 (MIS 5e) | CAV7-1_218 | -12.32 | -6.07 | 24.65 |
| Speleothem 1 (MIS 5e) | CAV7-1_219 | -12.06 | -5.84 | 24.89 |
| Speleothem 1 (MIS 5e) | CAV7-1_220 | -12.12 | -5.81 | 24.92 |
| Speleothem 1 (MIS 5e) | CAV7-1_221 | -12.30 | -5.98 | 24.75 |
| Speleothem 1 (MIS 5e) | CAV7-1_222 | -12.10 | -6.18 | 24.54 |
| Speleothem 1 (MIS 5e) | CAV7-1_223 | -12.06 | -6.36 | 24.35 |
| Speleothem 1 (MIS 5e) | CAV7-1_224 | -12.14 | -5.90 | 24.83 |
| Speleothem 1 (MIS 5e) | CAV7-1_225 | -12.09 | -6.29 | 24.43 |
| Speleothem 1 (MIS 5e) | CAV7-1_226 | -12.07 | -6.30 | 24.42 |
| Speleothem 1 (MIS 5e) | CAV7-1_227 | -12.16 | -6.13 | 24.59 |
| Speleothem 1 (MIS 5e) | CAV7-1_228 | -12.04 | -6.13 | 24.59 |
| Speleothem 1 (MIS 5e) | CAV7-1_229 | -12.00 | -5.88 | 24.85 |
| Speleothem 1 (MIS 5e) | CAV7-1_230 | -12.00 | -6.25 | 24.47 |
| Speleothem 1 (MIS 5e) | CAV7-1_231 | -12.06 | -6.27 | 24.45 |
| Speleothem 1 (MIS 5e) | CAV7-1_232 | -12.06 | -6.18 | 24.54 |
| Speleothem 1 (MIS 5e) | CAV7-1_233 | -12.02 | -6.05 | 24.67 |
| Speleothem 1 (MIS 5e) | CAV7-1_234 | -12.01 | -5.73 | 25.00 |
| Speleothem 1 (MIS 5e) | CAV7-1_235 | -12.06 | -6.07 | 24.65 |
| Speleothem 1 (MIS 5e) | CAV7-1_236 | -11.84 | -5.96 | 24.77 |
| Speleothem 1 (MIS 5e) | CAV7-1_237 | -11.82 | -6.32 | 24.39 |
| Speleothem 1 (MIS 5e) | CAV7-1_238 | -11.80 | -6.12 | 24.60 |
| Speleothem 1 (MIS 5e) | CAV7-1_239 | -11.63 | -5.65 | 25.09 |
| Speleothem 1 (MIS 5e) | CAV7-1_240 | -11.83 | -6.03 | 24.69 |
| Speleothem 1 (MIS 5e) | CAV7-1_241 | -11.91 | -5.98 | 24.75 |
| Speleothem 1 (MIS 5e) | CAV7-1_242 | -11.64 | -6.13 | 24.59 |
| Speleothem 1 (MIS 5e) | CAV7-1_243 | -11.51 | -5.98 | 24.75 |
| Speleothem 1 (MIS 5e) | CAV7-1_244 | -11.67 | -6.25 | 24.47 |
| Speleothem 1 (MIS 5e) | CAV7-1_245 | -11.63 | -6.28 | 24.44 |
| Speleothem 1 (MIS 5e) | CAV7-1_246 | -11.55 | -5.93 | 24.80 |
| Speleothem 1 (MIS 5e) | CAV7-1_247 | -11.62 | -5.80 | 24.93 |
| Speleothem 1 (MIS 5e) | CAV7-1_248 | -11.55 | -6.25 | 24.47 |
| Speleothem 1 (MIS 5e) | CAV7-1_249 | -11.52 | -6.01 | 24.71 |
| Speleothem 1 (MIS 5e) | CAV7-1_250 | -11.38 | -5.97 | 24.76 |
| Speleothem 1 (MIS 5e) | CAV7-1_251 | -11.30 | -5.98 | 24.75 |
| Speleothem 1 (MIS 5e) | CAV7-1_252 | -11.43 | -5.87 | 24.86 |
| Speleothem 1 (MIS 5e) | CAV7-1_253 | -11.33 | -5.95 | 24.78 |
| Speleothem 1 (MIS 5e) | CAV7-1_254 | -11.38 | -5.86 | 24.87 |
| Speleothem 1 (MIS 5e) | CAV7-1_255 | -11.33 | -5.54 | 25.20 |
| Speleothem 1 (MIS 5e) | CAV7-1_256 | -11.36 | -5.74 | 24.99 |
| Speleothem 1 (MIS 5e) | CAV7-1_257 | -11.29 | -6.13 | 24.59 |
| Speleothem 1 (MIS 5e) | CAV7-1_258 | -11.64 | -6.07 | 24.65 |
| Speleothem 1 (MIS 5e) | CAV7-1_259 | -11.65 | -6.11 | 24.61 |
| Speleothem 1 (MIS 5e) | CAV7-1_260 | -11.42 | -6.04 | 24.69 |
| Speleothem 1 (MIS 5e) | CAV7-1_261 | -11.43 | -6.22 | 24.50 |
| Speleothem 1 (MIS 5e) | CAV7-1_262 | -11.41 | -6.19 | 24.53 |
| Speleothem 1 (MIS 5e) | CAV7-1_263 | -11.39 | -6.31 | 24.41 |
| Speleothem 1 (MIS 5e) | CAV7-1_264 | -11.42 | -6.07 | 24.65 |
| Speleothem 1 (MIS 5e) | CAV7-1_265 | -11.64 | -5.90 | 24.83 |
| Speleothem 1 (MIS 5e) | CAV7-1_266 | -11.52 | -5.91 | 24.82 |
| Speleothem 1 (MIS 5e) | CAV7-1_267 | -11.92 | -5.98 | 24.74 |
| Speleothem 1 (MIS 5e) | CAV7-1_268 | -12.18 | -5.97 | 24.76 |
| Speleothem 1 (MIS 5e) | CAV7-1_269 | -12.24 | -6.28 | 24.44 |
| Speleothem 1 (MIS 5e) | CAV7-1_270 | -12.07 | -6.66 | 24.05 |
| Speleothem 1 (MIS 5e) | CAV7-1_271 | -11.18 | -6.08 | 24.64 |
| Speleothem 1 (MIS 5e) | CAV7-1_272 | -11.54 | -6.14 | 24.58 |
| Speleothem 1 (MIS 5e) | CAV7-1_273 | -12.02 | -6.45 | 24.26 |
| Speleothem 1 (MIS 5e) | CAV7-1_274 | -11.92 | -6.37 | 24.34 |
| Speleothem 1 (MIS 5e) | CAV7-1_275 | -11.81 | -6.29 | 24.43 |
| Speleothem 1 (MIS 5e) | CAV7-1_276 | -11.70 | -6.10 | 24.62 |
| Speleothem 1 (MIS 5e) | CAV7-1_277 | -11.76 | -5.96 | 24.76 |
| Speleothem 1 (MIS 5e) | CAV7-1_278 | -12.06 | -6.16 | 24.56 |
| Speleothem 1 (MIS 5e) | CAV7-1_279 | -12.49 | -6.23 | 24.49 |
| Speleothem 1 (MIS 5e) | CAV7-1_280 | -12.37 | -6.19 | 24.53 |
| Speleothem 1 (MIS 5e) | CAV7-1_281 | -12.35 | -5.74 | 24.99 |
| Speleothem 1 (MIS 5e) | CAV7-1_282 | -12.03 | -5.61 | 25.12 |
| Speleothem 1 (MIS 5e) | CAV7-1_283 | -12.10 | -5.56 | 25.18 |
| Speleothem 1 (MIS 5e) | CAV7-1_284 | -11.84 | -5.97 | 24.75 |
| Speleothem 1 (MIS 5e) | CAV7-1_285 | -11.74 | -5.79 | 24.94 |
| Speleothem 1 (MIS 5e) | CAV7-1_286 | -12.16 | -5.84 | 24.89 |
| Speleothem 1 (MIS 5e) | CAV7-1_287 | -11.48 | -5.97 | 24.75 |
| Speleothem 1 (MIS 5e) | CAV7-1_288 | -11.97 | -6.04 | 24.68 |
| Speleothem 1 (MIS 5e) | CAV7-1_289 | -11.98 | -6.51 | 24.20 |
| Speleothem 1 (MIS 5e) | CAV7-1_290 | -11.73 | -5.91 | 24.82 |
| Speleothem 1 (MIS 5e) | CAV7-1_291 | -11.92 | -6.49 | 24.22 |
| Speleothem 1 (MIS 5e) | CAV7-1_292 | -11.86 | -6.29 | 24.43 |
| Speleothem 1 (MIS 5e) | CAV7-1_293 | -11.69 | -6.11 | 24.61 |
| Speleothem 1 (MIS 5e) | CAV7-1_294 | -11.94 | -6.36 | 24.35 |
| Speleothem 1 (MIS 5e) | CAV7-1_295 | -11.69 | -6.41 | 24.30 |
| Speleothem 2 (Holocene) | CAV5-3_1 | -1.89 | -5.25 | 26.50 |
| Speleothem 2 (Holocene) | CAV5-3_2 | -3.27 | -4.97 | 26.78 |
| Speleothem 2 (Holocene) | CAV5-3_3 | -2.57 | -4.77 | 26.99 |
| Speleothem 2 (Holocene) | CAV5-3_4 | -2.60 | -5.59 | 26.15 |
| Speleothem 2 (Holocene) | CAV5-3_5 | -3.40 | -5.27 | 26.48 |
| Speleothem 2 (Holocene) | CAV5-3_6 | -4.06 | -5.12 | 26.64 |
| Speleothem 2 (Holocene) | CAV5-3_7 | -4.39 | -5.13 | 26.63 |
| Speleothem 2 (Holocene) | CAV5-3_8 | -1.23 | -5.04 | 26.71 |
| Speleothem 2 (Holocene) | CAV5-3_9 | -0.75 | -4.91 | 26.85 |
| Speleothem 2 (Holocene) | CAV5-3_10 | -1.59 | -5.10 | 26.66 |
| Speleothem 2 (Holocene) | CAV5-3_11 | -2.32 | -4.73 | 27.03 |
| Speleothem 2 (Holocene) | CAV5-3_12 | -1.82 | -4.75 | 27.01 |
| Speleothem 2 (Holocene) | CAV5-3_13 | -2.51 | -5.18 | 26.57 |
| Speleothem 2 (Holocene) | CAV5-3_16 | -1.68 | -4.84 | 26.92 |
| Speleothem 2 (Holocene) | CAV5-3_17 | -1.73 | -4.81 | 26.96 |
| Speleothem 2 (Holocene) | CAV5-3_18 | -1.54 | -4.83 | 26.93 |
| Speleothem 2 (Holocene) | CAV5-3_19 | -2.51 | -4.61 | 27.16 |
| Speleothem 2 (Holocene) | CAV5-3_20 | -1.82 | -5.01 | 26.74 |
| Speleothem 2 (Holocene) | CAV5-3_21 | -1.42 | -5.05 | 26.70 |
| Speleothem 2 (Holocene) | CAV5-3_22 | -1.57 | -4.62 | 27.14 |
| Speleothem 2 (Holocene) | CAV5-3_23 | -1.60 | -4.89 | 26.87 |
| Speleothem 2 (Holocene) | CAV5-3_24 | -1.34 | -4.70 | 27.07 |
| Speleothem 2 (Holocene) | CAV5-3_25 | -1.66 | -4.93 | 26.83 |
| Speleothem 2 (Holocene) | CAV5-3_26 | -1.91 | -4.65 | 27.12 |
| Speleothem 2 (Holocene) | CAV5-3_27 | -1.90 | -4.99 | 26.76 |
| Speleothem 2 (Holocene) | CAV5-3_28 | -1.64 | -4.79 | 26.97 |
| Speleothem 2 (Holocene) | CAV5-3_29 | -1.98 | -4.76 | 27.01 |
| Speleothem 2 (Holocene) | CAV5-3_30 | -0.33 | -4.39 | 27.39 |
| Speleothem 2 (Holocene) | CAV5-3_31 | -0.38 | -4.36 | 27.42 |
| Speleothem 2 (Holocene) | CAV5-3_32 | -0.84 | -4.74 | 27.02 |
| Speleothem 2 (Holocene) | CAV5-3_34 | -1.07 | -4.52 | 27.25 |
| Speleothem 2 (Holocene) | CAV5-3_36 | -0.60 | -4.41 | 27.37 |
| Speleothem 2 (Holocene) | CAV5-3_37 | -0.80 | -4.80 | 26.96 |
| Speleothem 2 (Holocene) | CAV5-3_39 | -1.02 | -4.58 | 27.19 |
| Speleothem 2 (Holocene) | CAV5-3_40 | -1.97 | -4.55 | 27.21 |
| Speleothem 2 (Holocene) | CAV5-3_42 | -3.69 | -5.42 | 26.32 |
| Speleothem 2 (Holocene) | CAV5-3_44 | -2.04 | -4.92 | 26.84 |
| Speleothem 2 (Holocene) | CAV5-3_45 | -3.03 | -4.93 | 26.83 |
| Speleothem 2 (Holocene) | CAV5-3_46 | -3.45 | -5.01 | 26.75 |
| Speleothem 2 (Holocene) | CAV5-3_47 | -5.10 | -5.37 | 26.38 |
| Speleothem 2 (Holocene) | CAV5-3_49 | -4.71 | -5.42 | 26.32 |
| Speleothem 2 (Holocene) | CAV5-3_50 | -3.78 | -5.50 | 26.24 |
| Speleothem 2 (Holocene) | CAV5-3_53 | -5.69 | -5.36 | 26.38 |
| Speleothem 2 (Holocene) | CAV5-3_54 | -6.48 | -5.25 | 26.50 |
| Speleothem 2 (Holocene) | CAV5-3_56 | -5.82 | -5.35 | 26.40 |
| Speleothem 2 (Holocene) | CAV5-3_57 | -4.20 | -5.05 | 26.70 |
| Speleothem 2 (Holocene) | CAV5-3_58 | -4.24 | -5.69 | 26.05 |
| Speleothem 2 (Holocene) | CAV5-3_59 | -4.73 | -5.28 | 26.47 |
| Speleothem 2 (Holocene) | CAV5-3_60 | -4.85 | -5.48 | 26.26 |
| Speleothem 2 (Holocene) | CAV5-3_61 | -4.65 | -5.56 | 26.17 |
| Speleothem 2 (Holocene) | CAV5-3_62 | -3.46 | -4.72 | 27.05 |
| Speleothem 2 (Holocene) | CAV5-3_63 | -3.69 | -5.09 | 26.67 |

Supplementary Table S4: Isotopic values for the two speleothems from Mora Cavorso.

**REFERENCES FOR SUPPLEMENTARY MATERIAL**

1 Venturo, D. & D’Onghia, P. La Necropoli Neolitica di Contrada Galliano, Palagiano (Taranto) in *Preistoria e Protostoria della Puglia* (ed. Radina, F.) 297-307 (Istituto Italiano di Preistoria e Protostoria, 2017).

2 Rolfo, M.F., Achino, K. & Silvestri, L. Mora Cavorso Cave: a collective underground burial in Neolithic central Italy in *Current Approaches to Collective Burials in the Late European Prehistory* (ed. Tomé, T. et al.) 33-40 (Archaeopress, 2017).

3 Scorrano, G., Rolfo, M.F., Brilli, M., Martínez-Labarga, C. & Rickards, O. Stable Isotope Analysis of Human and Faunal Remains from an Early Neolithic Italian Site: Mora Cavorso (Rome) in *Biological and cultural heritage of the Central-Southern Italian population through 30 thousand years. EPIC (Eredità della Popolazione dell'Italia Centro-Meridionale)* (ed. Rickards, O. & Sarti, L.) 153-170 (Universitalia, 2016).

4 Scorrano, G. et al. Effect Of Neolithic Transition On An Italian Community: Mora Cavorso (Jenne, Rome). *Archaeological and Anthropological Sciences* **11,** 1443–1459 (2019).

5 Radina, F. & Savino, M.L. Grotta Nisco (Cassano delle Murge-Bari), una necropoli dell’età del Rame. Lo studio di ‘ambiente 1’ e ‘ambiente 5’ in Papers in Italian Archaeology VII. The Archaeology of Death (ed. Herring, E. & O’Donoghue, E.) 443-447 (Archaeopress, 2018).

6 Venturo, D., Martinelli, M.C., Mossa, A.M. & Sublimi Saponetti, S. La Necropoli Eneolitica di Grotta Nisco in *Atti della XLIII Riunione Scientifica dell’Istituto Italiano di Preistoria e Protostoria*. 335-343 (Istituto Italiano di Preistoria e Protostoria, 2011).

7 Ceccaroni, E., Cosentino, S., Mangolini, D., Mieli, G. Pratovecchio (Celano, Prov. L’Aquila). Notiziario di Preistoria e Protostoria 48-50 (Istituto Italiano di Preistoria e Protostoria, 2014)

8 Cosentino, S., Mieli, G. & Ciarico, A. Aspetti e Problematiche dell’età del rame in Abruzzo alla luce di recenti scoperte. *Rivista Scienze Preistoriche* **LVII**, 139-166 (2007).

9 Silvestrini, M., Cazzella, A., Chilleri, F. & Pacciani, E. Antropologia e Paletnologia: una collaborazione per lo Studio della Necropoli Di Fontenoce (Recanati), Area Guzzini in *Atti della XLIII Riunione Scientifica dell'Istituto Italiano di Preistoria.* 387-395 (Istituto Italiano di Preistoria e Protostoria, Florence, 2011).

10 Volante, N. Spaccasasso Cave: a complex Copper age funerary contest on the Uccellina Mountains (Alberese – Grosseto) in *Biological and cultural heritage of the Central-Southern Italian population through 30 thousand years. EPIC (Eredità della Popolazione dell'Italia Centro-Meridionale)* (ed. Rickards, O. & Sarti, L) 93-104 (Universitalia, 2016).

11 Volante, N. & Sarti, L. Caves and shelters in the Uccellina Mountains (Alberese – Grosseto) – Funerary practices and rituals during the Bronze and Copper Ages at Grotta dello Scoglietto and Buca di Spaccasasso in *Papers in Italian Archaeology VII, The Archaeology of Death* (ed. Herring, E. & O’Donoghue, E.) 399-409 (National University of Ireland, 2018).

12 Anzidei, A.P. L’abitato Eneolitico di Osteria del Curato-Via Cinquefrondi: Nuovi Dati sulle Facies Archeologiche di Laterza e Ortucchio nel Territorio di Roma in *Atti della XL Riunione Scientifica dell’Istituto Italiano di Preistoria e Protostoria*, *2007.* (Istituto Italiano Preistoria e Protostoria, 2008)

13 Bailo Modesti, G & Salerno, A. Pontecagnano II, 5 La Necropoli Eneolitica, L'età del Rame in Campania nei villaggi dei morti. (Università degli studi di Napoli L'Orientale, 1998)

14 Anzidei, A.P. et al. L'età del rame nel territorio di Roma: rituali funerari nelle necropoli e nelle aree di abitato in *Biological and cultural heritage of the Central-Southern Italian population through 30 thousand years. EPIC (Eredità della Popolazione dell'italia Centro-Meridionale)* (eds. Rickards, O. & Sarti, L.) 15-34 (Universitalia, 2016).

15 De Angelis, F., Di Giannantonio, S., Scorrano, G., Catalano, P. & Rickards, O. An integrated approach to subsistence of the Eneolithic communities of Via CasettaMistici and Osteria del Curato – via Cinquefrondi in *Biological and cultural heritage of the Central-Southern Italian population through 30 thousand years. EPIC (Eredità della Popolazione dell'Italia Centro-Meridionale)* (eds Rickards, O. & Sarti, L.) 107-123 (Universitalia, 2016).

16 Bietti Sestieri, A.M. & Gianni, A. L'insediamento Eneolitico di Piscina di Torre Spaccata. La Campagna di Scavo -Relazione Preliminare in *Territorio di Roma* 142-154 (1984).

17 Angle, M. et al. Seppellimenti: tombe e fosse rituali nel sito preistorico di Pantano Borghese (Montecompatri, Roma) in *Atti dell’ottavo Incontro di Studi sul Lazio e la Sabina* 215-229 (Quasar, 2011).

18 Anzidei, A.P. et al. Il Gaudo a Sud del Tevere: Abitati e Necropoli dall’area Romana in *Atti della XLIII Riunione Scientifica dell’Istituto Italiano di Preistoria e Protostoria.* 309–321 (Istituto Italiano Preistoria e Protostoria, 2011).
